# DNA damage repair gene signature model for predicting prognosis and chemotherapy outcomes in lung squamous cell carcinoma

**DOI:** 10.1186/s12885-022-09954-x

**Published:** 2022-08-08

**Authors:** Xinshu Wang, Zhiyuan Huang, Lei Li, Guangxue Wang, Lin Dong, Qinchuan Li, Jian Yuan, Yunhui Li

**Affiliations:** 1 Jinzhou Medical University, Shanghai East Hospital, 200120 Shanghai, China; 2 Research Center for Translational Medicine, East Hospital, Tongji University School of Medicine, Shanghai, 200120 China; 3 Department of Cardiothoracic Surgery, East Hospital, Tongji University School of Medicine, Shanghai, 200120 China; 4 Department of Biochemistry and Molecular Biology, Tongji University School of Medicine, Shanghai, 200120 China; 5 Ji’an Hospital, Shanghai East Hospital, Ji’an, 343000 China

**Keywords:** LUSC, DNA damage repair genes, Risk model, Prognosis, Drug sensitivity

## Abstract

**Background:**

Lung squamous cell carcinoma (LUSC) is prone to metastasis and likely to develop resistance to chemotherapeutic drugs. DNA repair has been reported to be involved in the progression and chemoresistance of LUSC. However, the relationship between LUSC patient prognosis and DNA damage repair genes is still unclear.

**Methods:**

The clinical information of LUSC patients and tumour gene expression level data were downloaded from the TCGA database. Unsupervised clustering and Cox regression were performed to obtain molecular subtypes and prognosis-related significant genes based on a list including 150 DNA damage repair genes downloaded from the GSEA database. The coefficients determined by the multivariate Cox regression analysis and the expression level of prognosis-related DNA damage repair genes were employed to calculate the risk score, which divided LUSC patients into two groups: the high-risk group and the low-risk group. Immune viability, overall survival, and anticarcinogen sensitivity analyses of the two groups of LUSC patients were performed by Kaplan–Meier analysis with the log rank test, ssGSEA and the pRRophetic package in R software. A time-dependent ROC curve was applied to compare the survival prediction ability of the risk score, which was used to construct a survival prediction model by multivariate Cox regression. The prediction model was used to build a nomogram, the discriminative ability of which was confirmed by C-index assessment, and its calibration was validated by calibration curve analysis. Differentially expressed DNA damage repair genes in LUSC patient tissues were retrieved by the Wilcoxon test and validated by qRT–PCR and IHC.

**Result:**

LUSC patients were separated into two clusters based on molecular subtypes, of which Cluster 2 was associated with worse overall survival. A prognostic prediction model for LUSC patients was constructed and validated, and a risk score calculated based on the expression levels of ten DNA damage repair genes was employed. The clinical utility was evaluated by drug sensitivity and immune filtration analyses. Thirteen-one genes were upregulated in LUSC patient samples, and we selected the top four genes that were validated by RT–PCR and IHC.

**Conclusion:**

We established a novel prognostic model based on DNA damage repair gene expression that can be used to predict therapeutic efficacy in LUSC patients.

**Supplementary Information:**

The online version contains supplementary material available at 10.1186/s12885-022-09954-x.

## Background

Lung cancer is one of the most common malignant tumours in the world and has the greatest morbidity among all cancers. Lung cancer has become the leading cause of death from malignant tumours in China's urban population [1]. Most cases of lung cancer are non-small-cell lung cancer (NSCLC) [2]. NSCLCs account for approximately 80% of lung cancers, of which approximately 30% are LUSCs [3, 4]. Although many effective therapies have been applied, including surgery, chemotherapy, radiotherapy and targeted therapy, the prognosis of LUSC patients remains poor [5]. It is estimated that more than 60% of clinical stage I and II LUSC patients die 5 years after surgery due to relapse. Furthermore, approximately 75% of the patients have stage III or stage IV disease at diagnosis, and only 5% of these patients survive 5 years after surgery [6]. Chemotherapy with platinum therapy are currently used as basic treatments for patients with LUSC, but chemoresistance is a major obstacle leading to clinical failure [6]. Thus, it is necessary to identify novel molecular indicators in LUSC to calculate survival and identify chemoresistance in LUSC patients.

DNA damage develops in various kinds of cells during life. Cells have a DNA repair mechanism to avoid the fatal effect of DNA damage [7]. If the repair mechanism does not work properly, it leads to genome instability, cell apoptosis, cell cycle arrest, and even tumorigenesis [8]. Many kinds of DNA repair gene mutations exist in lung squamous cell cancer [9–11]. DNA damage repair is implicated not only in regulating the development of LUSC but also in resistance to chemoradiotherapy [12]. For instance, Ji W et al. evaluated the sensitivity of *BRCA1-* and *BRCA2-* deficient NSCLC cells to PARP inhibitors. However, few studies have concentrated on the relationships between DNA damage repair genes and the outcomes of LUSC patients.

In the present study, prognostic predictors were identified by performing Cox regression analysis of DNA repair genes. Risk scores were calculated based on the level of ten DNA damage repair genes related to LUSC patient prognosis. According to the expression levels of the ten genes and other clinical factors, we constructed a nomogram and model for prognosis prediction. We hope this research will identify potential molecular targets for predicting the prognosis and chemotherapy response of LUSC patients.

## Method

### Consensus clustering of DNA repair genes

The LUSC tissue data were clustered into k (2 to 9) groups by the ConsensuClusterPlus package in R software based on DNA repair genes. The k value was optimized according to the unsupervised clustering method, and LUSC cancer tissues showed consistent clustering. Two subgroups were obtained and verified by PCA. The survival of patients was compared by Kaplan–Meier analysis.

### Acquisition of DNA damage repair genes and clinical information of LUSC patients from the TCGA dataset

The DNA damage repair genes and the clinical information of the patients from whom the LUSC samples were derived were downloaded from the TCGA database. The information can be found in additional file Table S1. In total, 504 lung squamous cell cancer tissues were included in this study. A list including 150 DNA damage repair genes was downloaded from the hallmark gene set of the GSEA database to screen the gene expression matrix.

### Screening of differentially expressed DNA damage repair genes

The expression levels of DNA damage repair genes were compared by the Wilcoxon rank-sum test between the normal and tumour groups. The screening criteria were FDR (false discovery rate) < 0.05 and log_2_|fold change|> 1. The results of the differential DNA repair gene analysis are presented as volcano plots, heatmaps and box.

### Construction of the prognostic model

First, univariate Cox regression with the Wald χ^2^ test was used to establish the relationship between overall survival (OS) and DNA damage repair genes in LUSC patient tumour tissue. DNA repair genes with *p* values calculated by the Wald χ^2^ test less than 0.05 were considered statistically significant. According to the median expression level of DNA damage repair genes, the patients were divided into two groups: high and low expression groups. The overall survival of the two groups was analysed by the log-rank test, and survival curves were drawn. The multivariate Cox regression model was constructed by applying all the statistically significant variables in the univariate Cox regression. It was optimized by the AIC value in a stepwise algorithm. Then, a risk score based on the significant prognosis-related DNA damage repair genes was developed for LUSC patients: ($\mathrm{riskscore}={h}_{0}(\mathrm{t})\mathrm{exp}({\sum }_{j=1}^{n}{\mathrm{Coef}}_{j}\times {\mathrm{X}}_{j})$, where n is the quantity of sorted genes, h_0_(t) is the baseline risk function, Coef_j_ is the coefficient of each DNA repair gene, and X_j_ is the relative expression level of each DNA damage repair gene. The survival of LUSC patients with different risk scores was evaluated with prognostic hazard curves. Then, significant prognosis-related DNA damage repair genes were employed to construct a prognostic model with other clinical factors by multivariate Cox regression analysis. The predictive ability of the risk score and other clinical features were evaluated by time-dependent receiver operating characteristic (ROC) curve and area under the curve (AUC) analyses. The survival ROC package in R software was applied to draw the ROC curve. The AUC value, which indicates the sensitivity and specificity of the predictive indicators, varied from 0.5 to 1. The predictive ability of prognostic indicators increases with increasing AUC. The prognostic prediction model was ultimately developed into a nomogram, the calibration of which was measured with a calibration curve, and the discriminative ability was measured by C-index analysis.

### External validation of the risk score

To validate the prognostic predictive value of the risk score calculated based on the prognosis-related DNA repair genes, a gene expression level data matrix of lung cancer tissues with corresponding patient clinical data was downloaded from the GEO database (GSE31210). The risk score was calculated based on the formula constructed by the TCGA database. The prognosis-predicting ability of the risk score was estimated by time-dependent ROC curve analysis. According to the median risk score, the lung cancer patients in the GSE31210 dataset were divided into two groups: a high-risk group and a low-risk group. Kaplan–Meier curves of the two groups were drawn and compared by the log-rank test. Subsequently, the prognostic value of the risk score was estimated by univariate Cox proportional hazard regression. Furthermore, multivariate Cox proportional hazard regression revealed the risk score as an independent prognostic predictor.

### Immune and DNA repair genes in LUSC

Single-sample gene set enrichment analysis was performed by the "GSVA" package in R software with the method “ssGSEA” to calculate the infiltration scores of 16 types of immune cells. The infiltration scores of each tumour sample from LUSC patients in the high-risk group and low-risk group were calculated and compared by the Wilcoxon rank sum test. The immune infiltration scores of each type of immune cell and patient group were displayed as a box plot, as were the activities of 13 immune-related pathways (see additional file Table S2) [13, 14].

### Anticancer Agent Sensitivity Analysis

The IC50 values of six kinds of anticancer agents (etoposide, imatinib, methotrexate, rapamycin, vinorelbine, and vorinostat) were analysed in each lung squamous carcinoma sample. The pRRophetic package [15] in R software was applied to calculate the IC50 of each drug on the Genomics of Drug Sensitivity in Cancer website [16]. The half maximal inhibitory concentrations of drugs were compared between the high groups and low groups by the Wilcoxon rank-sum test.

### Real-Time Quantitative PCR

Total RNA from each specimen was purified by TRIzol (Invitrogen, USA). Then, RNA was transcribed into cDNA (complementary DNA) by the PrimeScript® RT Reagent Kit with gDNA (genomic DNA) Eraser (Takara, Japan). Real-time quantitative PCR was performed using a SYBR green master mix kit (ABI technology, USA). The QuantStudio System (Q6, Applied Biosystems, USA) was used to perform RT–qPCR. All samples were normalized to endogenous GAPDH (glyceraldehyde-3-phosphate dehydrogenase) with 2^−△△Ct^ algorithms. GenScript company (China) provided the primers for each gene.

### Immunohistochemistry

The LUSC tissue microarrays were incubated with antibodies (anti-RAE1, anti-POLR2H, anti-RAD51, anti-ZWINT and anti-RFC4) for immunohistochemical staining. The intensity and extent of staining were taken into consideration by the scoring system. Staining intensity was classified as 0 (negative), 1 (weak), 2 (moderate), or 3 (strong). The IHC score result was stratified as follows: 0 to 1, negative (-); 2 to 4, weakly positive (+ +); 5 to 8, moderately positive (+ +), and 9 to 12, strongly positive (+ + +).

## Result

### Molecular subgroups of LUSC clustered based on DNA damage repair genes

**Fig. 1:**
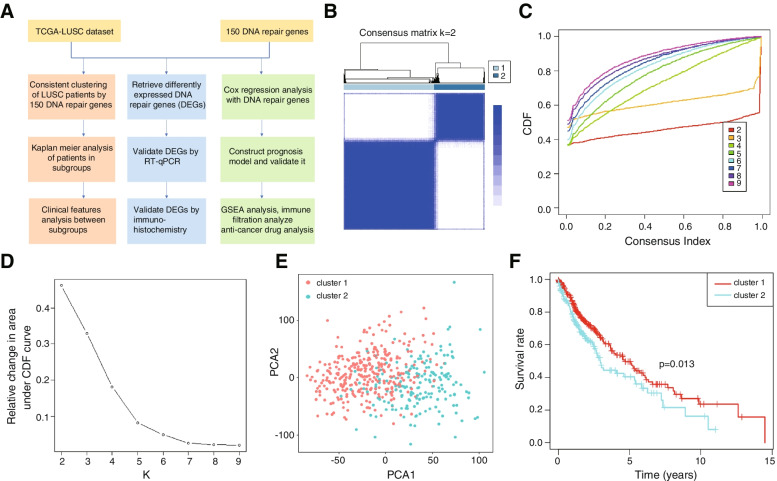
Stratify LUSC samples into two clusters with different prognoses. **A** Flow chart of the research. **B** The ConsensusClusterPlus package in R was applied to stratify LUSC samples into two clusters with different prognoses. **C** Consensus clustering matrix for k = 2. **D** Relative change in the area under the CDF curve. **E** PCA of the expression profile of DNA repair genes in Clusters 1 and 2. **F** Kaplan–Meier curves of patient overall survival between Cluster 1 and Cluster 2

An analysis flowchart is shown in Fig. 1A. To investigate the characteristics of DNA damage repair genes in LUSC, we divided the LUSC samples from TCGA into subgroups based on the expression of 150 genes related to DNA damage repair, which were downloaded from the GSEA website by the R package ConsensusClusterPlus. Clustering stability was analysed from k = 2 to 9 for the TCGA datasets, and k = 2 was identified as the best value, showing expression similarity of the DNA damage repair-related genes. The subgroups were divided into Cluster 1 and Cluster 2 and the division of lung squamous carcinoma samples by DNA repair genes showed a good differentiation effect validated by PCA analysis (Fig. 1B-E). Survival analysis also showed a significant difference between these 2 subgroups (*P* value = 0.013) (Fig. 1F). These results suggested that two groups of lung squamous carcinoma patients stratified by concensus cluster were different in clinical characters.

### Determination of the prognostic significance of DNA damage repair-related genes

**Fig. 2:**
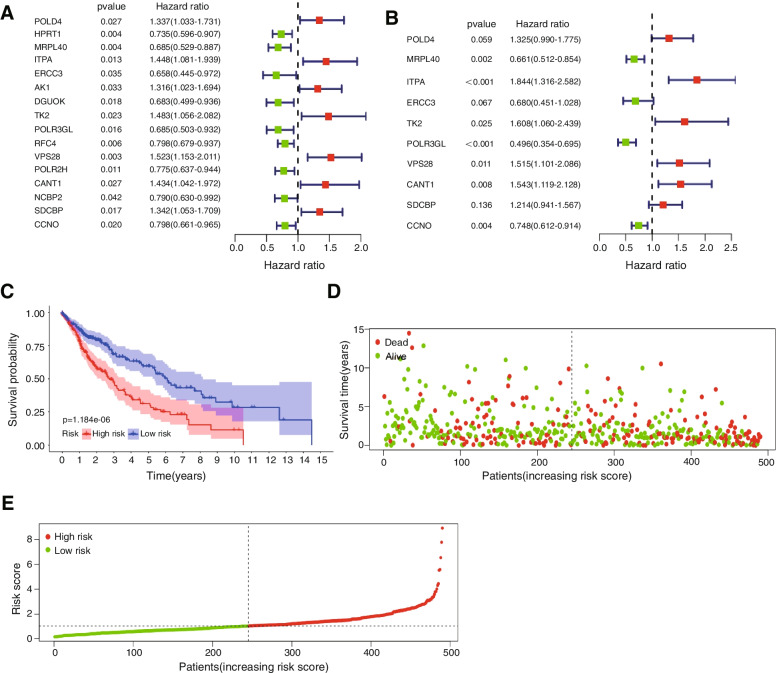
Forest plot of and Kaplan–Meier curves for high- and low-risk group patients. **A** Forest plot of 16 prognosis-related DNA repair genes identified by univariate Cox regression. **B** Forest plot of 10 prognosis-related genes identified by the multivariate Cox regression model after optimization based on the AIC value. **C** KM curve of overall survival for LUSC patients in the high-risk and low-risk groups stratified by the median risk score. **D** Risk score scatter plot for patients who survived and those who died. Patients who died are presented as red dots. Patients who survived are presented as green dots. **E** The individual inflection point of the risk score curve is displayed by a dotted line. Patients were divided into low-risk and high-risk groups by the median risk score. Red dots represent patients with high risk. Green dots represent patients with low risk

The expression profile dataset, which included 504 LUSC samples, was obtained from the TCGA database. Clinical information of these 504 patients was listed in Table 1. First, univariate Cox proportional hazard regression with the Wald χ^2^ test was used to identify 16 DNA damage repair genes (*POLD4*, *HPRT1*, *MRPL40*, *ITPA*, *ERCC3*, *AK1*, *DGUOK*, *TK2*, *POLR3GL*, *RFC4*, *VPS28*, *POLR2H*, *CANT1*, *NCBP2*, *SDCBP*, and *CCNO*). The expression level of these genes was significantly correlated with the overall survival of LUSC patients (Fig. 2A). Moreover, multivariate Cox regression models were constructed using these genes. The model with the lowest AIC value was selected for further analysis to avoid overfitting. After optimization based on the AIC value, ten DNA repair genes (*POLD4*, *MRPL40*, *ITPA*, *ERCC3*, *TK2*, *POLR3GL*, *VPS28*, *CANT1*, *SDCBP*, and *CCNO*) were preserved in the last multivariate Cox regression model and had potential to be prognostic factors (Fig. 2B, additional file Table S3).

**Table 1:** Clinical information of training cohort and validation cohort

		**TCGA-training cohort**	**GEO-validation cohort**
Gender	Male	373	105
	Female	131	121
Age	< 60	91	96
	> = 60	404	130
Pathological stage	Stage I	245	168
	Stage II	163	58
	Stage III	85	-
	Stage IV	7	-
	unknow	4	-
Survival Status	Alive	304	191
	Dead	200	35
T stage	T1 stage	114	-
	T2 stage	295	-
	T3 stage	71	-
	T4 stage	24	-
N stage	N0 stage	320	-
	N1 stage	133	-
	N2 stage	40	-
	N3 stage	5	-
	unknow	6	-
M stage	M0	414	-
	M1	7	-
	unknow	83	-

Based on their relationship with LUSC patient survival (HR > 1), six genes (*SDCBP*, *POLD4*, *VPS28*, *CANT1*, *TK2*, and *ITPA*) were considered risk factors, but the other four genes (*POLR3GL*, *MRPL40*, *ERCC3*, and *CCNO*) played protective roles (HR < 1). Ultimately, the risk scores of the patients were calculated based on the expression of these ten significant prognosis-related DNA damage repair genes and their coefficients in the multivariate Cox regression model. The median risk score was used to classify the LUSC patients into a high-risk group and a low-risk group. Overall survival was significantly different between the two groups of patients (median time = 2.64 years vs. 6.16 years, log rank *p* value < 0.001, Fig. 2C).

Prognostic hazard curves for the LUSC patients showed the distribution of the risk score. The survival time and risk score results were visualized with a scatter plot to display the survival time of each patient with the corresponding risk score (Fig. 2D-E). The results revealed that patients with higher risk scores had shorter survival.

### Analysis of the relationship between TNM stage and the expression of significant DNA damage repair-related genes

**Fig. 3:**
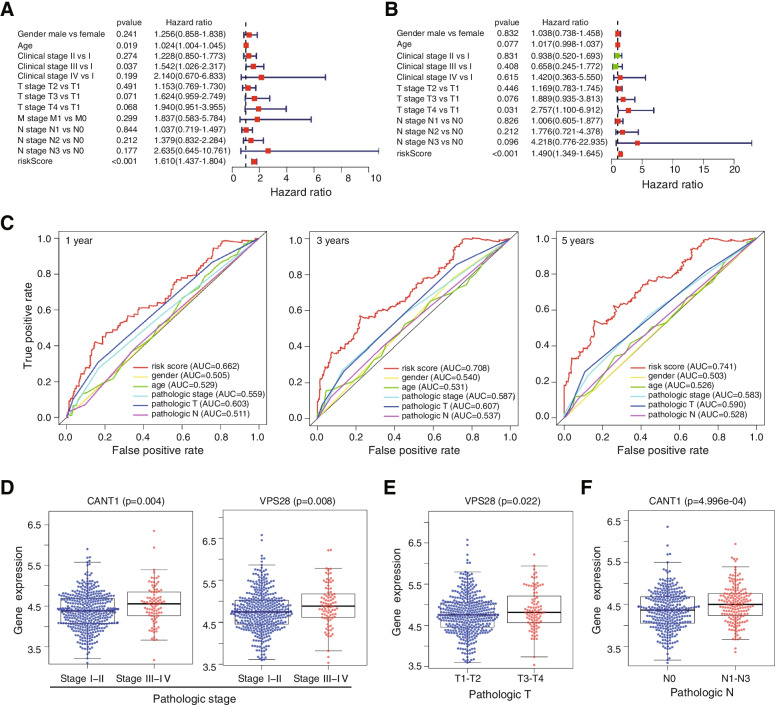
Forest plots for the risk score and other clinical features. **A** Forest plot for risk score and clinical features in the univariate Cox proportional risk regression model. **B** Forest plot for the risk score and clinical features in the multivariate Cox proportional risk regression model. **C** ROC curves for evaluating the ability of the factors to discriminate 1-year, 3-year, and 5-year survival. The risk score was estimated to better predict prognosis than other clinical features. AUC: area under the curve. The discriminative ability increased with increasing AUC. **D-F** Box plot displaying the relationship between prognostic DNA repair gene expressions and clinical features

The risk score and TNM stage were used to perform univariate Cox regression analysis. Pathologic stage (stage III vs. stage I HR = 1.542, *P* value = 0.037) and the risk score (HR = 1.610, *P* value < 0.001) were correlated with the overall survival (OS) of LUSC patients (Fig. 3A). Multivariate Cox regression analysis of these clinical features showed that T stage (HR = 2.757, *P* value = 0.031) and the risk score (HR = 1.490, *P *value < 0.001) were independent risk factors for survival (Fig. 3B).

To assess the difference between risk scores and other prognosis-related clinical features, time-dependent ROC curves were constructed for 1-year, 3-year and 5-year survival. Moreover, we used the area under the curve (AUC) values to assess the ability of each prognostic predictor to discriminate between patients who survived and those who died. The AUC of the risk score was larger than that of age, stage and T stage at 1 year, 3 years and 5 years, which indicated that the risk score was a better prognostic predictor than other clinical features (risk score AUC = 0.662, 0.708, 0.741 for 1 year, 3 years and 5 years, respectively) (Fig. 3C).

We applied a t test or Kruskal–Wallis test to assess the correlation between the DNA damage repair genes and TNM stage. The expression levels of *CANT1* and *VPS28* were increased in advanced stage compared with stage I-II to stage III-IV disease (*P* value = 0.004 and *P* value = 0.008) (Fig. 3D). In T3-T4 stage patients, the expression level of *VPS28* was higher than that in early T stage patients (*P* value = 0.02), implying its dangerous role in the development of LUSC (Fig. 3E). Furthermore, the expression level of *CANT1* was higher in N1-N3 LUSC tissues than in N0 stage LUSC tissues, which was determined based on the distribution of *CANT1* expression levels between N0 stage and N1-N3 stage tissues (Fig. 3F). Thus, we conclude that the disruption of DNA damage repair might be responsible for the poor prognosis of patients with LUSC.

### External validation of the risk score

**Fig. 4:**
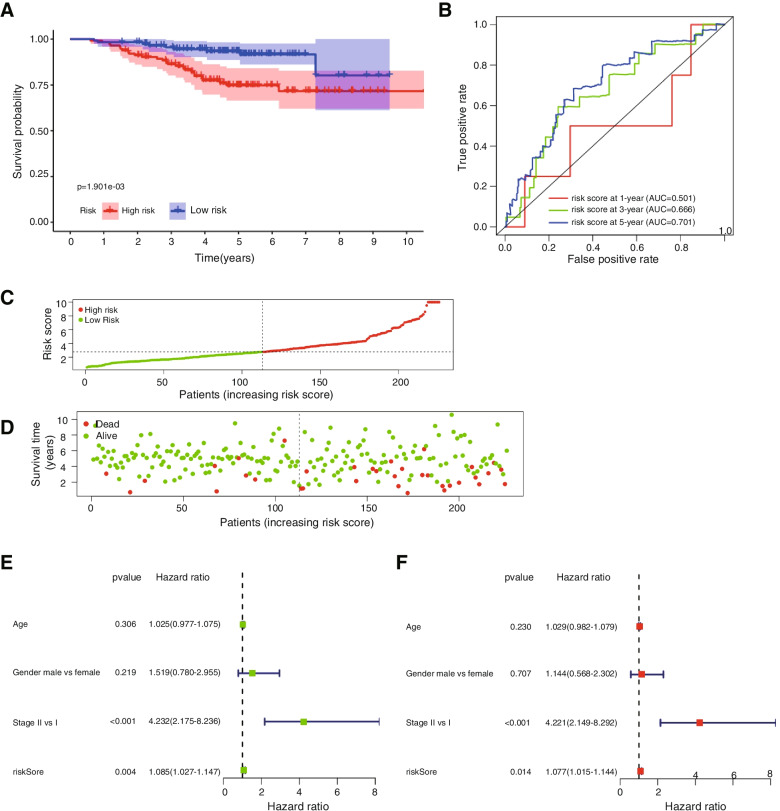
Validation of the risk score based on significant prognosis-related DNA repair genes in GSE31210. **A** Kaplan–Meier analysis of patients from the high-risk group and the low-risk group. **B** ROC curve analysis of the risk score at 1 year, 3 years and 5 years. **C** Scatter plot showing the risk scores of high-risk group patients and low-risk group patients. The individual inflection points of the risk score curve is displayed by dotted lines. **D** The risk scores and corresponding survival times and survival states of different patients. **E**–**F** Forest plot for the univariate and multivariate Cox regression analyses of prognostic indicators, including the risk score

The RNA-seq data and clinical data of the lung cancer tissues were downloaded from the GEO database (GSE31210). Totally 226 patients were involved into research, after the filtration of patients without survival time. The detail of clinical information was presented in Table 1. The risk score was calculated with the formula based on patient data from the TCGA database and the expression level of prognostic genes in the GSE31210 dataset. Patients in GSE31210 were divided into a high-risk group and a low-risk group according to the median risk score. The difference in overall survival between the high-risk group and the low-risk group was statistically significant (Fig. 4A) (log-rank test *P* value = 1.901^−03^). The ability of the risk score to predict prognosis was estimated by the area under the curve (AUC) of the time-dependent ROC curve (Fig. 4B). Prognostic hazard curves were drawn to analyse the utility of the prognostic DNA repair genes (Fig. 4C-D). The survival is much higher in both high and low risk groups in the validation GEO data (GSE31210) set as compared to the discovery TCA set. It is perhaps due to only early stages (stage I and stage II) cancer patients’ data in this validation (Table 1). Actually, early LUSC detection leads to the better survival outcomes of patients. We also analysed the hazard ratio of the risk score using univariate and multivariate Cox regression (Fig. 4E-F). Similar results were derived from the GSE31210 cohort and the TCGA LUSC cohort. Therefore, the risk scores were correlated with the overall survival (OS) of LUSC patients, and univariate or multivariate Cox regression analyses verified DNA repair-related gene-based model could be served as an independent prognostic indicator of LUSC.

### Establishment and validation of the nomogram

**Fig. 5:**
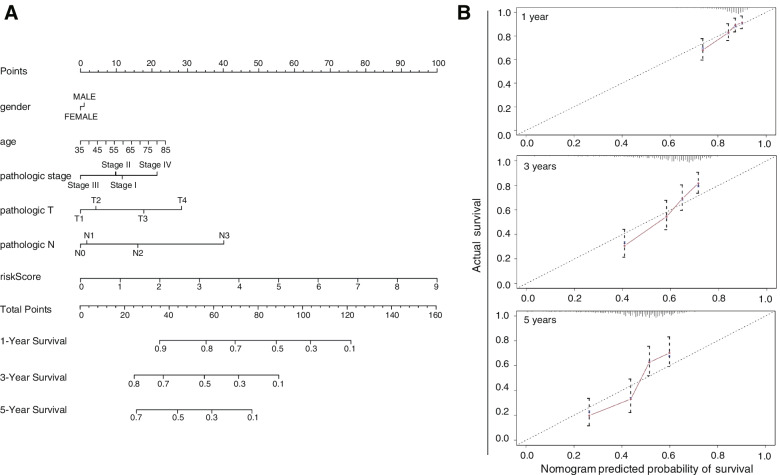
Prediction model constructed for LUSC patients. **A** The nomogram considering sex, age, clinical stage, T stage, N stage and the risk score based on ten prognosis-related DNA repair genes predicted the 1-year, 3-year and 5-year survival of LUSC patients. **B** Calibration curves of the ability of the nomogram to predict prognosis at 1-year, 3-year and 5-year

A nomogram was generated to utilize the constructed prognostic model for LUSC patients. We selected tumour stage, N stage, T stage, risk score, sex and age to establish the nomogram (Fig. 5A). The discriminatory ability of the nomogram was estimated based on the C-index, which varied from 0.5 to 1. The discriminatory ability increased with increasing C-index. The results showed that the C-index of the constructed nomogram was 0.669. Furthermore, the calibration curves of the nomogram at 1 year, 3 years and 5 years are displayed in Fig. 5B. The closer the calibration curve is to the diagonal line, the more precise the calibration is. Taken together, these C-index and calibration curve data suggest that the nomogram can be used to predict the prognosis of LUSC patients.

### Evaluation of cancer therapy agents in different risk groups

**Fig. 6:**
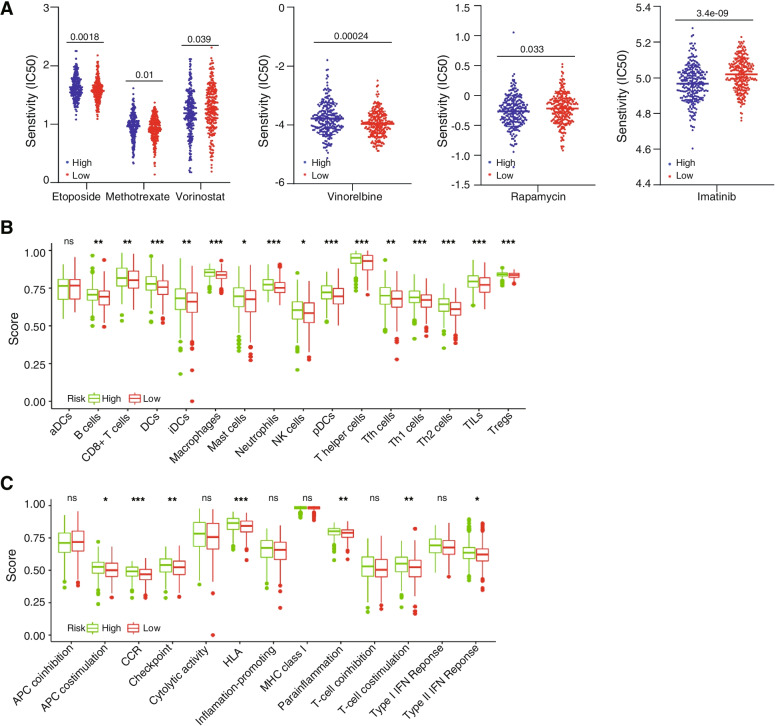
Evaluation of cancer therapy agents in the high-risk and low-risk patient groups. **A** The half maximal inhibitory concentration (IC50) values for each of 6 anticancer drugs (etoposide, imatinib, methotrexate, rapamycin, vinorelbine, and vorinostat) were compared between the high-risk group and the low-risk group, and the results are displayed in box plots. Each dot represents the estimated IC50 value of the corresponding drug in the LUSC sample. The higher the IC50 is, the less sensitive the LUSC sample is to the drug. **B** The scores of 16 immune cells. **C** The scores of 13 immune-related functions. DCs dendritic cells, iDCs immature DCs, pDCs plasmacytoid dendritic cells, TIL tumour-infiltrating lymphocyte, CCR cytokine–cytokine receptor, APC antigen-presenting cells. Adjusted *P* values are shown as follows: ns, not significant; *P < 0.05; **P < 0.01; ***P < 0.001

pRRophetic was applied to estimate the sensitivity of the high-risk group and the low-risk group of LUSC patients to anticancer agents, including etoposide, imatinib, methotrexate, rapamycin, vinorelbine and vorinostat. The analysis of anticancer agent sensitivity demonstrated that etoposide, methotrexate and vinorelbine had higher IC50 levels in the high-risk group, implying that low-risk group patients is more sensitive to the three drugs. In contrast, the IC50 values of imatinib, vorinostat and rapamycin were higher in the low-risk group, which indicated that high-risk group patients is more sensitive to the three drugs (Fig. 6A).

DNA damage repair defects will lead to increased genomic instability and tumour tumorigenesis, which may activate the tumour immune response. The infiltration scores of 16 kinds of immune cells and the enrichment scores of 13 corresponding immune functions were estimated by the “ssGSEA” method, which is provided in the “GSVA” R package. The analysis results revealed that 15 kinds of immune cell subpopulations (B cells, NK cells, macrophages, mast cells, Tregs, T helper cells, TILs, Th1 cells, Th2 cells, Tfh cells, CD8 + T cells, DCs, iDCs, neutrophils, and pDCs) had lower scores in the low-risk group than in the high-risk group (Fig. 6B). Furthermore, we also found that the scores of 7 immune functions were also significantly lower in the low-risk group, including T-cell costimulation, parainflammation, APC costimulation, CCR, checkpoint, HLA and type II IFN response (Fig. 6C). These results suggest that immunological functions are more active in the high-risk group than in the low-risk group, and these functions may be related to the expression level of DNA damage repair genes. Combining DDR-targeting drugs and tumourimmunotherapy to treat LUSC holds wide application prospects.

### Differentially expressed DNA repair genes

**Fig. 7:**
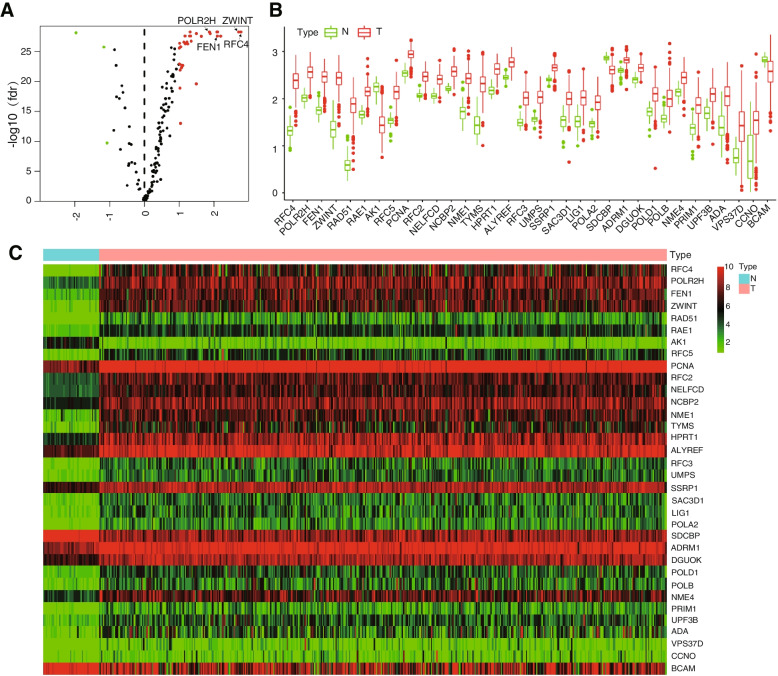
The expression of differentially expressed DNA repair genes. **A** Volcano plot of the DNA repair genes. The vertical axis represents the false discovery rate. The horizontal axis represents the fold change in the expression level between the cancer group and the normal group. Red dots represent upregulated genes. Green dots represent downregulated genes. **B** Differentially expressed DNA repair genes are presented in a box plot. (**C**) The heatmap displays the distribution of differentially expressed DNA repair genes among the cancer and normal tissues. Green represents downregulated genes. Red represents upregulated genes

Differentially expressed DNA repair genes were retrieved from the gene expression profile dataset downloaded from the TCGA database. The dataset included 49 normal lung tissue samples and 501 lung squamous cancer tissue samples. Thirty-four differentially expressed DNA repair genes (DEGs) were ultimately retrieved. Thirty-one genes were upregulated and three genes were downregulated in the tumour group compared with the normal group. The genes are displayed in additional file Table S4. The DEGs are presented in volcano plots, box plots and heatmaps (Fig. 7A-C). Most of the DNA repair genes were upregulated in the tumour group, which indicates that cancer cells might have better DNA repair abilities that help them survive in a hostile environment.

### The expression levels of differentially expressed DNA repair genes in LUSC tumour tissues

**Fig. 8:**
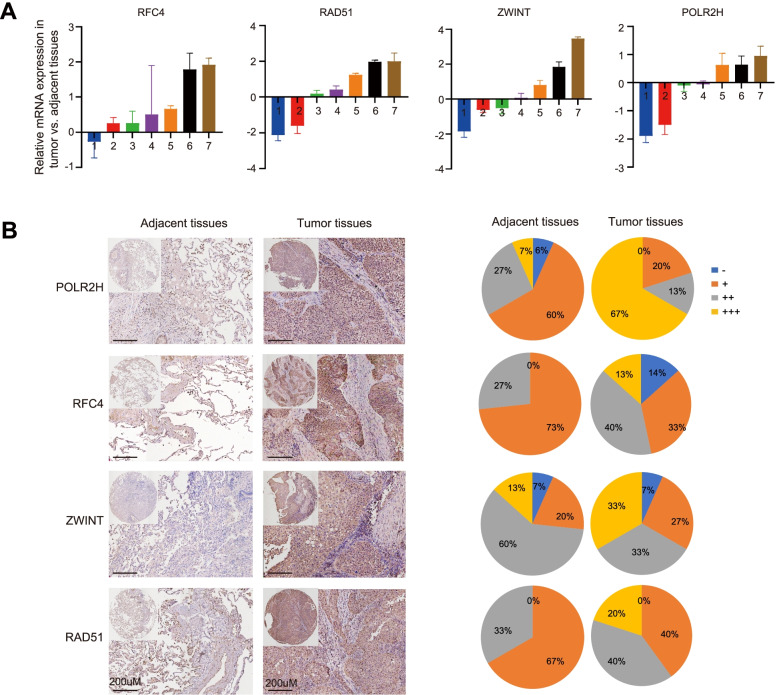
The different expression levels of DNA repair genes between LUSC tumor tissues and adjacent normal tissues. **A**Total RNA was isolated from 7 pairs of clinical LUSC tumor tissue and adjacent normal tissue. Relative mRNA expression was analyzed by qPCR. Each bar is the log2 value of the ration of 4 genes between tumor and adjacent normal tissues. **B** IHC analysis of the indicated genes in LUSC tumor tissues and adjacent normal tissues

To further verify the differentially expressed DNA repair genes in LUSC, we used real-time quantitative PCR (qRT–PCR) and immunohistochemistry (IHC) to analyse the four genes (*POLR2H*, *RFC4*, *ZWINT*, and *RAD51*) that had the most significantly different differences in expression. The qRT–PCR results showed that the four genes were upregulated in the tumour group compared with adjacent normal tissues, which was consistent with the results of the differential expression analysis of the RNA-seq data from TCGA (Fig. 8A). To confirm the RNA-seq results, the four genes were validated by immunohistochemical staining in lung squamous cancer tissue microarrays. *POLR2H*, *RAD51*, *ZWINT* and *RFC4* were expressed at higher levels in LUSC tissues than in adjacent normal tissues (Fig. 8B). These genes should be validated in larger-scale clinical studies in the future. The molecular biological function of these genes deserves further exploration.

## Discussion

Genomic DNA damage caused by smoking or exposure to harmful chemical and physical factors is believed to be the first stage of carcinogenesis in lung cancer. It has been reported that the process of cancer development can be affected greatly by the expression level of DNA repair genes in tumour tissues, which can help sustain the stability of the cancer cell genome [17]. A case–control study showed that lung cancer patients had a reduced DNA repair capacity (DRC) [18]. On the other hand, another case–control study pointed out that lung cancer patients with higher DNA repair capacity had elevated chemoresistance [19]. These previous reports found similar to our research showing that DNA repair genes may have both protective and unfavourable effects in the development of LUSC in specific patients [20]. In light of the important role that DNA repair genes play in the origination and development of lung cancer, we performed bioinformatics analysis to identify significant prognosis-related DNA repair genes in LUSC.

Our research uncovered and evaluated the prognostic value of ten DNA repair genes (*POLD4*, *MRPL40*, *ITPA*, *ERCC3*, *TK2*, *POLR3GL*, *VPS28*, *CANT1*, *SDCBP*, and *CCNO*). The function of these genes in lung adenocarcinoma has been reported in previous studies. *POLD4* has an important role in genomic instability, double-stranded DNA breaks (DSBs) and lung cancer. *POLD4* decreases the intrinsically high induction of γ-H2AX, a marker of DSBs [21]. The expression levels of *TK2* were significantly associated with prognosis in lung cancer tissues. The levels of *TK2* were higher, and the prognosis of LUSC patients was better [22]. Higher *CANT1* expression was closely related to the TN stage. High expression levels and promoter demethylation of *CANT1* were related to worse prognosis in LUSC [23, 24]. Other papers have also shown that *CCNO* is a key protein in lung physiology, and *CCNO* mutations result in lung disease [25]. Moreover *CCNO* upregulation is significantly associated with reduced overall survival in lung cancer patients [26]. In our study, prognostic predictors were identified via Cox regression analysis based on DNA repair genes. The risk scores of each LUSC patient were calculated based on the expression levels of the ten prognosis-related DNA repair genes. Overall, the prognostic model based on these ten genes was a useful tool for predicting the prognosis of LUSC patients.

Many DNA repair-related genes have been proven to be involved in the progression of distinct kinds of cancer. Such genes have been applied as signatures for determining the prognosis of cancer. Wang et al. identified eleven genes that were able to predict the survival of patients with colon cancer [27]. Hu et al. constructed a prognostic prediction model based on 13 DNA repair genes for lung adenocarcinoma patients [28]. Twenty-eight DNA repair genes related to the prognosis of patients with ovarian cancer were identified, and some of them were applied to construct a prognostic model of ovarian cancer [29]. A set of seven genes were used to predict the survival of patients with hepatocellular carcinoma [30]. Liu et al. discovered that a nine DNA repair gene set had prominent clinical implications for prognosis evaluation and could predict the survival of patients with endometrial carcinoma. Similarly, a DNA repair gene signature was applied to establish a prognostic nomogram for predicting the biochemical recurrence-free survival of prostate cancer patients [31]. However, the relationship between the expression level of DNA repair genes and LUSC patients remains unclear. In this study, we created a novel prognostic prediction model based on DNA repair genes for lung squamous carcinoma. Our model provides clinicians with a way to evaluate the survival of lung squamous carcinoma patients.

Chemotherapy with cisplatin is currently used as basic treatments for patients with LUSC, but chemoresistance is a major obstacle leading to clinical failure [32, 33]. Actually, LUSC is the least sensitive to chemotherapy compared with other types of NSCLC. It is an important question how to select suitable chemotherapeutic drug for patients in order to obtain more benefit. DNA repair has been reported to be involved in the progression and chemoresistance of LUSC. In our study, prognostic predictors were identified by performing Cox regression analysis of DNA repair genes. Patients with a low-risk score may be more sensitive to etoposide, methotrexate and vinorelbine, and high-risk group patients is more sensitive to the imatinib, vorinostat and rapamycin, suggesting that different groups of patients have different sensitivity to drugs. Therefore, we hoped that we established this novel prognostic model based on DNA damage repair gene expression that can be used to predict therapeutic efficacy with LUSC patients.

## Conclusion

In this study, a novel prognostic model based on DNA repair genes was constructed for lung squamous carcinoma patients. Our model is able to effectively predict the sensitivity of anticancer therapy. Furthermore, this study provides potential independent biomarkers that could be applied in the clinic.

## Supplementary Information

**Additional file 1:** **Table S1.** Clinical information. **Table S2.** Marker For ssGSEA. **Table S3.** Prognosis related DNA repair genes. **Table S4.** Differently expressed genes.

## Data Availability

The datasets generated and analysed during the current study are available in the TCGA and GEO repository. https://portal.gdc.cancer.gov/repository https://www.ncbi.nlm.nih.gov/geo/query/acc.cgi?acc=GSE31210
