# Supplementary material for: DNA damage repair gene signature model for predicting prognosis and chemotherapy outcomes in lung squamous cell carcinoma
Source: BMC Cancer. 2022 Aug 8;22:866. doi: 10.1186/s12885-022-09954-x (PMC9361681; doi:10.1186/s12885-022-09954-x)
Supplement: Supplementary file 1 — Additional file 1: Table S1. Clinical information. Table S2. Marker For ssGSEA. Table S3. Prognosis related DNA repair genes. Table S4. Differently expressed genes. [file 12885_2022_9954_MOESM1_ESM.zip › Table S1. clinical information_ESM.pdf]

Additional file table S1 clinical information

Id	futime	fustat	gender	race	age	pathologic stage	pathologic T	pathologic M	pathologic N	newevent	histological type
TCGA-85-6561	395	0	MALE	WHITE	66	Stage IB	T2a	M0	NX	unknow	Lung Squamous Cell Carcinoma- Not Otherwise Specified (NOS)
TCGA-37-5819	103	0	MALE	WHITE	64	Stage IIIA	T2	M0	N2	unknow	Lung Squamous Cell Carcinoma- Not Otherwise Specified (NOS)
TCGA-77-8008	2639	1	MALE	unknow	68	Stage IB	T2	M0	N0	unknow	Lung Squamous Cell Carcinoma- Not Otherwise Specified (NOS)
TCGA-56-8083	150	0	MALE	WHITE	56	Stage IB	T2a	MX	N0	NO	Lung Squamous Cell Carcinoma- Not Otherwise Specified (NOS)
TCGA-56-A4ZJ	640	0	FEMALE	WHITE	75	Stage IA	T1a	M0	N0	NO	Lung Squamous Cell Carcinoma- Not Otherwise Specified (NOS)
TCGA-85-7710	42	0	FEMALE	WHITE	59	Stage IA	T1b	M0	N0	unknow	Lung Squamous Cell Carcinoma- Not Otherwise Specified (NOS)
TCGA-77-A5G1	4026	0	MALE	unknow	75	Stage IIIA	T3	M0	N1	YES	Lung Squamous Cell Carcinoma- Not Otherwise Specified (NOS)
TCGA-22-5480	2170	1	FEMALE	WHITE	66	Stage IA	T1b	M0	N0	unknow	Lung Squamous Cell Carcinoma- Not Otherwise Specified (NOS)
TCGA-85-7698	952	0	MALE	WHITE	48	Stage IA	T1b	M0	N0	unknow	Lung Squamous Cell Carcinoma- Not Otherwise Specified (NOS)
TCGA-63-A5MU		1	MALE	unknow	48	Stage IIB	T2b	M0	N1	YES	Lung Squamous Cell Carcinoma- Not Otherwise Specified (NOS)
TCGA-34-A5IX	1031	0	MALE	WHITE	80	Stage IIB	T3	M0	N0	NO	Lung Squamous Cell Carcinoma- Not Otherwise Specified (NOS)
TCGA-52-7811	266	1	MALE	WHITE	67	Stage IB	T2	M0	N0	unknow	Lung Squamous Cell Carcinoma- Not Otherwise Specified (NOS)
TCGA-58-8387	403	1	MALE	WHITE	60	Stage IIA	T2b	M0	N0	NO	Lung Squamous Cell Carcinoma- Not Otherwise Specified (NOS)
TCGA-21-5783	2680	1	MALE	WHITE	76	Stage IB	T2	M0	N0	unknow	Lung Squamous Cell Carcinoma- Not Otherwise Specified (NOS)
TCGA-85-A511	455	1	MALE	WHITE	62	Stage IIB	T2b	M0	N1	NO	Lung Squamous Cell Carcinoma- Not Otherwise Specified (NOS)
TCGA-94-8035	122	0	MALE	WHITE	64	Stage IIB	T3	MX	N0	NO	Lung Squamous Cell Carcinoma- Not Otherwise Specified (NOS)
TCGA-37-4129	242	0	FEMALE	WHITE	52	Stage IA	T1b	M0	N0	unknow	Lung Squamous Cell Carcinoma- Not Otherwise Specified (NOS)
TCGA-77-8153	1992	0	FEMALE	unknow	77	Stage IB	T2	M0	N0	NO	Lung Squamous Cell Carcinoma- Not Otherwise Specified (NOS)
TCGA-NC-A5HG	1575	0	MALE	WHITE	59	Stage IIIA	T2	M0	N2	NO	Lung Basaloid Squamous Cell Carcinoma
TCGA-77-8146	2183	0	MALE	unknow	72	Stage IA	T1	M0	N0	unknow	Lung Squamous Cell Carcinoma- Not Otherwise Specified (NOS)
TCGA-J1-A4AH	581	0	MALE	WHITE	70	Stage IIA	T2b	MX	N0	NO	Lung Squamous Cell Carcinoma- Not Otherwise Specified (NOS)
TCGA-94-7033	87	0	MALE	WHITE	73	Stage IB	T2	MX	N0	unknow	Lung Squamous Cell Carcinoma- Not Otherwise Specified (NOS)

TCGA-66-2787	1217	0	MALE	unknow	57	Stage IA	T1	M0	N0	unknow	Lung Squamous Cell Carcinoma- Not Otherwise Specified (NOS)
TCGA-63-A5MJ	1824	0	MALE	unknow	54	Stage IIB	T2	M0	N1	NO	Lung Squamous Cell Carcinoma- Not Otherwise Specified (NOS)
TCGA-18-3421	2645	0	MALE	WHITE	65	Stage IB	T2	M0	N0	unknow	Lung Squamous Cell Carcinoma- Not Otherwise Specified (NOS)
TCGA-21-1079	965	1	MALE	WHITE	71	Stage IIIA	T3	M0	N0	unknow	Lung Squamous Cell Carcinoma- Not Otherwise Specified (NOS)
TCGA-34-5231	1984	1	MALE	WHITE	72	Stage IA	T1	M0	N0	unknow	Lung Squamous Cell Carcinoma- Not Otherwise Specified (NOS)
TCGA-NC-A5HE	1949	0	MALE	WHITE	60	Stage IIB	T2	M0	N1	NO	Lung Squamous Cell Carcinoma- Not Otherwise Specified (NOS)
TCGA-34-2600	1874	1	FEMALE	WHITE	76	Stage IA	T1	M0	N0	unknow	Lung Squamous Cell Carcinoma- Not Otherwise Specified (NOS)
TCGA-66-2769	215	1	MALE	unknow	75	Stage IIIB	T4	M0	N0	unknow	Lung Squamous Cell Carcinoma- Not Otherwise Specified (NOS)
TCGA-18-4083	188	1	MALE	WHITE	63	Stage IIB	T2	M0	N1	unknow	Lung Squamous Cell Carcinoma- Not Otherwise Specified (NOS)
TCGA-33-AAS8	1114	1	FEMALE	BLACK OR AFRICAN AMERICAN	59	Stage IA	T1	MX	N0	NO	Lung Squamous Cell Carcinoma- Not Otherwise Specified (NOS)
TCGA-77-8130	2522	0	MALE	unknow	69	Stage IIB	T2	M0	N1	unknow	Lung Squamous Cell Carcinoma- Not Otherwise Specified (NOS)
TCGA-85-8352	161	1	MALE	WHITE	67	Stage IIIA	T3	M0	N1	unknow	Lung Squamous Cell Carcinoma- Not Otherwise Specified (NOS)
TCGA-85-A512	465	0	MALE	WHITE	46	Stage IIA	T1b	M0	N1	unknow	Lung Squamous Cell Carcinoma- Not Otherwise Specified (NOS)
TCGA-77-8145	212	1	MALE	unknow	73	Stage IIIB	T4	M0	N1	YES	Lung Squamous Cell Carcinoma- Not Otherwise Specified (NOS)
TCGA-58-8388	412	1	MALE	WHITE	60	Stage IB	T2a	M0	N0	NO	Lung Basaloid Squamous Cell Carcinoma
TCGA-18-3415	2803	1	MALE	unknow	77	Stage IB	T2	M0	N0	unknow	Lung Squamous Cell Carcinoma- Not Otherwise Specified (NOS)
TCGA-33-4582	3149	1	MALE	WHITE	55	Stage IA	T1	M0	N0	unknow	Lung Squamous Cell Carcinoma- Not Otherwise Specified (NOS)
TCGA-02-A52V	1335	1	FEMALE	BLACK OR AFRICAN AMERICAN	75	Stage II	T3	MX	N0	YES	Lung Squamous Cell Carcinoma- Not Otherwise Specified (NOS)
TCGA-77-7335	2133	1	FEMALE	WHITE	62	Stage IIIB	T4	M0	N2	unknow	Lung Squamous Cell Carcinoma- Not Otherwise Specified (NOS)
TCGA-18-4721	4694	0	MALE	WHITE	74	Stage IA	T1	M0	N0	unknow	Lung Squamous Cell Carcinoma- Not Otherwise Specified (NOS)
TCGA-96-8170	531	0	FEMALE	WHITE	75	Stage IIA	T1a	M0	N1	NO	Lung Squamous Cell Carcinoma- Not Otherwise Specified (NOS)
TCGA-52-7810	923	0	FEMALE	WHITE	60	Stage IIB	T3	M0	N0	unknow	Lung Squamous Cell Carcinoma- Not Otherwise Specified (NOS)
TCGA-NC-A5HR	905	0	FEMALE	WHITE	75	Stage IIA	T2a	M0	N1	NO	Lung Basaloid Squamous Cell Carcinoma
TCGA-39-5021	2086	1	MALE	WHITE	70	Stage IB	T2a	M0	N0	unknow	Lung Squamous Cell Carcinoma- Not Otherwise Specified (NOS)

TCGA-56-7582	601	0	MALE	WHITE	83	Stage IB	T2a	M0	N0	unknow	Lung Squamous Cell Carcinoma- Not Otherwise Specified (NOS)
TCGA-96-7545	1344	0	MALE	WHITE	73	Stage IA	T1	MX	N0	unknow	Lung Squamous Cell Carcinoma- Not Otherwise Specified (NOS)
TCGA-22-5474	445	1	MALE	WHITE	74	Stage IB	T2a	M0	N0	unknow	Lung Squamous Cell Carcinoma- Not Otherwise Specified (NOS)
TCGA-63-A5MI	1784	0	MALE	unknow	65	Stage IIIA	T2	M0	N2	NO	Lung Squamous Cell Carcinoma- Not Otherwise Specified (NOS)
TCGA-51-6867	1856	1	FEMALE	WHITE	72	Stage I	T1	M0	N0	unknow	Lung Squamous Cell Carcinoma- Not Otherwise Specified (NOS)
TCGA-66-2780	366	1	MALE	unknow	65	Stage IB	T2	M0	N0	unknow	Lung Squamous Cell Carcinoma- Not Otherwise Specified (NOS)
TCGA-85-A510	482	0	FEMALE	WHITE	74	Stage IIB	T2b	M0	N1	NO	Lung Squamous Cell Carcinoma- Not Otherwise Specified (NOS)
TCGA-90-7964	428	0	MALE	WHITE	70	Stage IB	T2a	MX	N0	unknow	Lung Squamous Cell Carcinoma- Not Otherwise Specified (NOS)
TCGA-56-8309	428	0	MALE	WHITE	66	Stage IA	T1b	MX	N0	NO	Lung Squamous Cell Carcinoma- Not Otherwise Specified (NOS)
TCGA-21-5786	661	0	MALE	WHITE	64	Stage IB	T2	M0	N0	unknow	Lung Squamous Cell Carcinoma- Not Otherwise Specified (NOS)
TCGA-85-8582	424	0	MALE	WHITE	49	Stage IA	T1a	M0	N0	unknow	Lung Squamous Cell Carcinoma- Not Otherwise Specified (NOS)
TCGA-98-A53B	61	1	MALE	WHITE	69	Stage IB	T2a	M0	N0	NO	Lung Squamous Cell Carcinoma- Not Otherwise Specified (NOS)
TCGA-66-2757	1338	1	FEMALE	unknow	65	Stage IA	T1	M0	N0	unknow	Lung Squamous Cell Carcinoma- Not Otherwise Specified (NOS)
TCGA-98-8022	933	1	MALE	WHITE	61	Stage IA	T1a	M0	N0	NO	Lung Squamous Cell Carcinoma- Not Otherwise Specified (NOS)
TCGA-85-7950	576	0	MALE	WHITE	46	Stage IB	T2a	M0	N0	unknow	Lung Squamous Cell Carcinoma- Not Otherwise Specified (NOS)
TCGA-66-2791	153	1	MALE	unknow	66	Stage IIIB	T2	M0	N3	unknow	Lung Squamous Cell Carcinoma- Not Otherwise Specified (NOS)
TCGA-98-A53H	618	0	FEMALE	WHITE	76	Stage IA	T1a	M0	N0	NO	Lung Squamous Cell Carcinoma- Not Otherwise Specified (NOS)
TCGA-56-8625	315	1	FEMALE	WHITE	66	Stage IIIA	T3	MX	N1	NO	Lung Squamous Cell Carcinoma- Not Otherwise Specified (NOS)
TCGA-34-8454	827	0	FEMALE	WHITE	62	Stage IIIA	T3	M0	N1	NO	Lung Squamous Cell Carcinoma- Not Otherwise Specified (NOS)
TCGA-70-6723	375	0	MALE	ASIAN	65	Stage IIA	T3	M0	N0	unknow	Lung Papillary Squamous Cell Caricnoma
TCGA-34-2604	958	1	FEMALE	WHITE	81	Stage IB	T2	M0	N0	unknow	Lung Squamous Cell Carcinoma- Not Otherwise Specified (NOS)
TCGA-46-3769	135	0	MALE	WHITE	57	unknow	T4	M0	N0	unknow	Lung Squamous Cell Carcinoma- Not Otherwise Specified (NOS)
TCGA-22-1012	429	1	FEMALE	WHITE	80	Stage IB	T2	M0	N0	unknow	Lung Squamous Cell Carcinoma- Not Otherwise Specified (NOS)
TCGA-21-1072	3016	0	MALE	WHITE	75	Stage IB	T2	M0	N0	unknow	Lung Squamous Cell Carcinoma- Not Otherwise Specified (NOS)
TCGA-22-4595	734	1	MALE	WHITE	57	Stage IIIA	T3	unknow	N2	unknow	Lung Squamous Cell Carcinoma- Not Otherwise Specified (NOS)

TCGA-34-5929	151	1	FEMALE	WHITE	78	Stage IB	T2	M0	N0	unknow	Lung Squamous Cell Carcinoma- Not Otherwise Specified (NOS)
TCGA-85-A4QQ	553	0	MALE	WHITE	68	Stage IB	T2a	M0	N0	unknow	Lung Squamous Cell Carcinoma- Not Otherwise Specified (NOS)
TCGA-NK-A5D1	511	0	MALE	WHITE	57	Stage IIA	T2a	M0	N1	YES	Lung Basaloid Squamous Cell Carcinoma
TCGA-63-6202		0	MALE	unknow	unk now	Stage IIA	T2	M0	N0	unknow	Lung Squamous Cell Carcinoma- Not Otherwise Specified (NOS)
TCGA-60-2724	717	0	MALE	WHITE	47	Stage IIIA	T3	M0	N1	unknow	Lung Squamous Cell Carcinoma- Not Otherwise Specified (NOS)
TCGA-34-5240	365	0	FEMALE	WHITE	73	Stage IIB	T2	M0	N1	unknow	Lung Basaloid Squamous Cell Carcinoma
TCGA-66-2773	92	1	MALE	unknow	69	Stage IB	T2	M0	N0	unknow	Lung Squamous Cell Carcinoma- Not Otherwise Specified (NOS)
TCGA-39-5039	544	1	MALE	WHITE	76	Stage IIA	T2b	M0	N0	unknow	Lung Squamous Cell Carcinoma- Not Otherwise Specified (NOS)
TCGA-60-2697	372	1	MALE	WHITE	41	Stage IIA	T2	M0	N2	unknow	Lung Squamous Cell Carcinoma- Not Otherwise Specified (NOS)
TCGA-63-7023		0	MALE	unknow	unk now	Stage IIA	T1	M0	N1	unknow	Lung Squamous Cell Carcinoma- Not Otherwise Specified (NOS)
TCGA-56-7822	532	1	MALE	BLACK OR AFRICAN AMERICAN	75	Stage IIB	T2b	M0	N1	unknow	Lung Squamous Cell Carcinoma- Not Otherwise Specified (NOS)
TCGA-77-8140	351	1	FEMALE	unknow	66	Stage IIB	T2	M0	N1	unknow	Lung Squamous Cell Carcinoma- Not Otherwise Specified (NOS)
TCGA-77-8128	1150	1	MALE	unknow	60	Stage IIIA	T2	M0	N2	unknow	Lung Squamous Cell Carcinoma- Not Otherwise Specified (NOS)
TCGA-22-5491	1713	1	MALE	WHITE	74	Stage IA	T1a	M0	N0	unknow	Lung Squamous Cell Carcinoma- Not Otherwise Specified (NOS)
TCGA-66-2783	759	0	MALE	unknow	67	Stage IIIB	T2	M0	N3	unknow	Lung Squamous Cell Carcinoma- Not Otherwise Specified (NOS)
TCGA-60-2707	667	1	MALE	unknow	70	Stage IB	T2	M0	N0	unknow	Lung Squamous Cell Carcinoma- Not Otherwise Specified (NOS)
TCGA-58-8392	501	1	MALE	WHITE	70	Stage IB	T2a	M0	N0	YES	Lung Squamous Cell Carcinoma- Not Otherwise Specified (NOS)
TCGA-66-2778	578	0	FEMALE	unknow	68	Stage IIIB	T2	M0	N3	unknow	Lung Squamous Cell Carcinoma- Not Otherwise Specified (NOS)
TCGA-34-8456	804	0	FEMALE	WHITE	71	Stage IIA	T2a	M0	N1	NO	Lung Squamous Cell Carcinoma- Not Otherwise Specified (NOS)
TCGA-66-2753	31	0	MALE	unknow	69	Stage IB	T2	M0	N0	unknow	Lung Squamous Cell Carcinoma- Not Otherwise Specified (NOS)
TCGA-98-A53C	822	0	FEMALE	WHITE	77	Stage IA	T1a	M0	N0	NO	Lung Squamous Cell Carcinoma- Not Otherwise Specified (NOS)
TCGA-33-AASL	826	1	FEMALE	BLACK OR AFRICAN AMERICAN	57	Stage IA	T1	MX	N0	YES	Lung Squamous Cell Carcinoma- Not Otherwise Specified (NOS)
TCGA-85-8277	307	1	MALE	WHITE	70	Stage IIIA	T3	M0	N1	unknow	Lung Squamous Cell Carcinoma- Not Otherwise Specified (NOS)

TCGA-63-7020		0	MALE	unknow	unk now	Stage IA	T1	M0	N0	unknow	Lung Squamous Cell Carcinoma- Not Otherwise Specified (NOS)
TCGA-22-5483	573	1	MALE	WHITE	74	Stage IIA	T1a	M0	N1	unknow	Lung Squamous Cell Carcinoma- Not Otherwise Specified (NOS)
TCGA-63-A5MH	2026	0	MALE	unknow	68	Stage IA	T1	M0	N0	NO	Lung Squamous Cell Carcinoma- Not Otherwise Specified (NOS)
TCGA-56-A4BY	543	1	MALE	WHITE	66	Stage IB	T2a	MX	N0	NO	Lung Squamous Cell Carcinoma- Not Otherwise Specified (NOS)
TCGA-52-7812	835	1	MALE	WHITE	68	unknow	T2	M0	N2	unknow	Lung Squamous Cell Carcinoma- Not Otherwise Specified (NOS)
TCGA-60-2708	2447	0	FEMALE	WHITE	64	Stage IIB	T2	M0	N1	unknow	Lung Squamous Cell Carcinoma- Not Otherwise Specified (NOS)
TCGA-85-7696	376	0	MALE	WHITE	64	Stage IA	T1	M0	N0	unknow	Lung Squamous Cell Carcinoma- Not Otherwise Specified (NOS)
TCGA-60-2709	1505	0	MALE	BLACK OR AFRICAN AMERICAN	69	Stage IB	T2	MX	N0	unknow	Lung Squamous Cell Carcinoma- Not Otherwise Specified (NOS)
TCGA-18-3416	973	1	MALE	unknow	83	Stage IIB	T2	M0	N1	unknow	Lung Squamous Cell Carcinoma- Not Otherwise Specified (NOS)
TCGA-90-A59Q	322	1	FEMALE	WHITE	61	Stage IIA	T2a	MX	N1	NO	Lung Squamous Cell Carcinoma- Not Otherwise Specified (NOS)
TCGA-85-8353	94	1	MALE	WHITE	72	Stage IIIA	T3	M0	N1	unknow	Lung Squamous Cell Carcinoma- Not Otherwise Specified (NOS)
TCGA-18-3406	371	1	MALE	WHITE	67	Stage IA	T1	M0	N0	unknow	Lung Squamous Cell Carcinoma- Not Otherwise Specified (NOS)
TCGA-85-8287	23	1	MALE	WHITE	72	Stage IA	T1b	M0	N0	NO	Lung Squamous Cell Carcinoma- Not Otherwise Specified (NOS)
TCGA-37-4133	238	0	MALE	WHITE	63	Stage IIIA	T4	M0	N0	unknow	Lung Squamous Cell Carcinoma- Not Otherwise Specified (NOS)
TCGA-58-A46M	1072	0	MALE	WHITE	61	Stage IIB	T2b	M0	N1	NO	Lung Squamous Cell Carcinoma- Not Otherwise Specified (NOS)
TCGA-56-8628	616	0	MALE	WHITE	78	Stage IA	T1b	MX	N0	NO	Lung Squamous Cell Carcinoma- Not Otherwise Specified (NOS)
TCGA-85-8666	475	0	MALE	WHITE	65	Stage IB	T2a	M0	N0	unknow	Lung Squamous Cell Carcinoma- Not Otherwise Specified (NOS)
TCGA-6A-AB49		1	FEMALE	BLACK OR AFRICAN AMERICAN	73	Stage IB	T2	MX	N0	NO	Lung Squamous Cell Carcinoma- Not Otherwise Specified (NOS)
TCGA-66-2768	61	0	MALE	unknow	57	Stage IIB	T2	M0	N1	unknow	Lung Squamous Cell Carcinoma- Not Otherwise Specified (NOS)
TCGA-58-A46J	2589	0	MALE	WHITE	64	Stage IIB	T2	M0	N1	NO	Lung Squamous Cell Carcinoma- Not Otherwise Specified (NOS)
TCGA-56-8082	455	0	FEMALE	WHITE	80	Stage IIA	T2b	MX	N0	NO	Lung Squamous Cell Carcinoma- Not Otherwise Specified (NOS)
TCGA-NC-A5HL	88	1	MALE	WHITE	73	Stage IIA	T2b	M0	N0	NO	Lung Squamous Cell Carcinoma- Not Otherwise Specified (NOS)
TCGA-56-8201	397	1	MALE	WHITE	74	Stage IIB	T3	MX	N0	NO	Lung Squamous Cell Carcinoma- Not Otherwise Specified (NOS)

TCGA-85-8350	683	0	MALE	WHITE	61	Stage IA	T1b	M0	N0	unknow	Lung Squamous Cell Carcinoma- Not Otherwise Specified (NOS)
TCGA-21-1083	1315	1	MALE	WHITE	75	Stage IA	T1	M0	N0	unknow	Lung Squamous Cell Carcinoma- Not Otherwise Specified (NOS)
TCGA-85-6560	364	0	MALE	WHITE	59	Stage IIA	T1b	M0	N1	unknow	Lung Squamous Cell Carcinoma- Not Otherwise Specified (NOS)
TCGA-94-8491	810	0	MALE	WHITE	73	Stage IIA	T2a	M0	N1	NO	Lung Squamous Cell Carcinoma- Not Otherwise Specified (NOS)
TCGA-33-4583	4601	1	MALE	WHITE	73	Stage IA	T1	M0	N0	unknow	Lung Squamous Cell Carcinoma- Not Otherwise Specified (NOS)
TCGA-22-4607	587	1	MALE	WHITE	75	Stage IB	T2a	M0	N0	unknow	Lung Squamous Cell Carcinoma- Not Otherwise Specified (NOS)
TCGA-85-6175	294	1	FEMALE	WHITE	63	Stage IIB	T3	M0	N0	unknow	Lung Squamous Cell Carcinoma- Not Otherwise Specified (NOS)
TCGA-22-4593	1067	1	MALE	WHITE	77	Stage IIA	T2b	M0	N0	unknow	Lung Squamous Cell Carcinoma- Not Otherwise Specified (NOS)
TCGA-66-2794	1645	0	MALE	unknow	64	Stage IIIB	T4	M0	N2	unknow	Lung Squamous Cell Carcinoma- Not Otherwise Specified (NOS)
TCGA-66-2759	762	0	MALE	unknow	66	Stage IIIA	T2	M0	N2	unknow	Lung Squamous Cell Carcinoma- Not Otherwise Specified (NOS)
TCGA-MF-A522	360	1	MALE	WHITE	54	Stage IB	T2a	MX	N0	NO	Lung Squamous Cell Carcinoma- Not Otherwise Specified (NOS)
TCGA-85-8052	734	0	MALE	WHITE	53	Stage IIB	T3	M0	N0	NO	Lung Squamous Cell Carcinoma- Not Otherwise Specified (NOS)
TCGA-85-8072	513	0	MALE	WHITE	60	Stage IA	T1a	M0	N0	unknow	Lung Squamous Cell Carcinoma- Not Otherwise Specified (NOS)
TCGA-46-6026	423	0	MALE	WHITE	81	Stage IIB	T2a	M0	N1	unknow	Lung Squamous Cell Carcinoma- Not Otherwise Specified (NOS)
TCGA-85-A53L	377	0	MALE	ASIAN	63	Stage IIA	T2b	M0	N0	unknow	Lung Squamous Cell Carcinoma- Not Otherwise Specified (NOS)
TCGA-63-7022		0	FEMALE	unknow	unknow	Stage IA	T1	M0	N0	unknow	Lung Squamous Cell Carcinoma- Not Otherwise Specified (NOS)
TCGA-56-5898	555	0	MALE	WHITE	69	Stage IA	T1b	M0	N0	unknow	Lung Squamous Cell Carcinoma- Not Otherwise Specified (NOS)
TCGA-77-A5G3	3576	0	MALE	unknow	63	Stage IIB	T2	M0	N1	NO	Lung Squamous Cell Carcinoma- Not Otherwise Specified (NOS)
TCGA-33-4589	47	1	FEMALE	WHITE	62	Stage IIB	T2	M0	N1	unknow	Lung Squamous Cell Carcinoma- Not Otherwise Specified (NOS)
TCGA-18-3408	2304	1	FEMALE	WHITE	77	Stage IB	T2	M0	N0	unknow	Lung Squamous Cell Carcinoma- Not Otherwise Specified (NOS)
TCGA-39-5031	833	0	FEMALE	unknow	76	Stage IA	T1a	M0	N0	unknow	Lung Squamous Cell Carcinoma- Not Otherwise Specified (NOS)
TCGA-79-5596		0	MALE	unknow	unknow	Stage IIIA	T3	M0	N1	unknow	Lung Squamous Cell Carcinoma- Not Otherwise Specified (NOS)
TCGA-02-A52Q	113	1	FEMALE	WHITE	44	Stage III	T3	MX	N1	YES	Lung Squamous Cell Carcinoma- Not Otherwise Specified (NOS)
TCGA-85-6798	195	1	MALE	WHITE	57	Stage IIIA	T3	M0	N1	unknow	Lung Squamous Cell Carcinoma- Not Otherwise Specified (NOS)

TCGA-66-2781	121	0	MALE	unknow	67	Stage IB	T2	M0	N0	unknow	Lung Squamous Cell Carcinoma- Not Otherwise Specified (NOS)
TCGA-56-A4BX	405	0	MALE	WHITE	70	Stage IIA	T2b	MX	N0	NO	Lung Squamous Cell Carcinoma- Not Otherwise Specified (NOS)
TCGA-56-6546		0	MALE	WHITE	67	Stage IIA	T2b	MX	N0	unknow	Lung Squamous Cell Carcinoma- Not Otherwise Specified (NOS)
TCGA-39-5040	519	1	MALE	WHITE	59	Stage IIIA	T2a	M0	N2	unknow	Lung Squamous Cell Carcinoma- Not Otherwise Specified (NOS)
TCGA-98-7454	256	0	MALE	WHITE	73	Stage IB	T2a	M0	N0	unknow	Lung Squamous Cell Carcinoma- Not Otherwise Specified (NOS)
TCGA-60-2715	1075	1	MALE	WHITE	51	Stage IA	T1	M0	N0	unknow	Lung Squamous Cell Carcinoma- Not Otherwise Specified (NOS)
TCGA-18-5592	1519	0	MALE	unknow	57	Stage IIB	T3	M0	N0	unknow	Lung Squamous Cell Carcinoma- Not Otherwise Specified (NOS)
TCGA-34-5241	515	1	MALE	WHITE	79	Stage IB	T2	M0	N0	unknow	Lung Squamous Cell Carcinoma- Not Otherwise Specified (NOS)
TCGA-43-A56V	366	0	MALE	BLACK OR AFRICAN AMERICAN	61	Stage IIIA	T2a	M0	N2	NO	Lung Squamous Cell Carcinoma- Not Otherwise Specified (NOS)
TCGA-56-8307	818	0	FEMALE	WHITE	55	Stage IIB	T3	M0	N0	NO	Lung Squamous Cell Carcinoma- Not Otherwise Specified (NOS)
TCGA-77-A5GF	839	0	MALE	unknow	70	Stage IIA	T2a	M0	N1	YES	Lung Squamous Cell Carcinoma- Not Otherwise Specified (NOS)
TCGA-43-5668	349	0	MALE	WHITE	78	Stage IIA	T1b	M0	N1	unknow	Lung Squamous Cell Carcinoma- Not Otherwise Specified (NOS)
TCGA-63-A5MT	498	0	MALE	unknow	74	Stage IIB	T3	M0	N0	YES	Lung Squamous Cell Carcinoma- Not Otherwise Specified (NOS)
TCGA-85-8580	445	0	FEMALE	WHITE	52	Stage IB	T2a	M0	N0	unknow	Lung Squamous Cell Carcinoma- Not Otherwise Specified (NOS)
TCGA-22-5492	506	1	FEMALE	unknow	73	Stage IIIA	T2a	M0	N2	unknow	Lung Squamous Cell Carcinoma- Not Otherwise Specified (NOS)
TCGA-51-4079	12	1	FEMALE	BLACK OR AFRICAN AMERICAN	73	Stage IB	T2	unknow	N0	unknow	Lung Squamous Cell Carcinoma- Not Otherwise Specified (NOS)
TCGA-56-8304	106	0	FEMALE	WHITE	73	Stage IA	T1b	MX	N0	NO	Lung Squamous Cell Carcinoma- Not Otherwise Specified (NOS)
TCGA-68-A59J	448	0	FEMALE	WHITE	74	Stage IB	T2a	MX	N0	NO	Lung Squamous Cell Carcinoma- Not Otherwise Specified (NOS)
TCGA-63-5128		1	MALE	unknow	unk now	Stage IB	T2	M0	N0	unknow	Lung Squamous Cell Carcinoma- Not Otherwise Specified (NOS)
TCGA-21-1080	3724	0	MALE	WHITE	66	Stage IB	T2	M0	N0	unknow	Lung Squamous Cell Carcinoma- Not Otherwise Specified (NOS)
TCGA-39-5034	1107	1	FEMALE	WHITE	73	Stage IIB	T3	M0	N0	unknow	Lung Squamous Cell Carcinoma- Not Otherwise Specified (NOS)
TCGA-77-7138	340	1	MALE	unknow	67	Stage IB	T2	M0	N0	unknow	Lung Squamous Cell Carcinoma- Not Otherwise Specified (NOS)
TCGA-58-A46K	1045	1	MALE	WHITE	59	Stage IIIA	T2	M0	N2	YES	Lung Basaloid Squamous Cell Carcinoma

TCGA-63-A5MN	345	1	FEMALE	unknow	78	Stage IIB	T3	M0	N0	YES	Lung Squamous Cell Carcinoma- Not Otherwise Specified (NOS)
TCGA-66-2790	699	0	MALE	unknow	72	Stage IIB	T2	M0	N1	unknow	Lung Squamous Cell Carcinoma- Not Otherwise Specified (NOS)
TCGA-85-7843	35	0	MALE	WHITE	50	Stage IIA	T2a	M0	N1	unknow	Lung Squamous Cell Carcinoma- Not Otherwise Specified (NOS)
TCGA-63-A5MB	3123	0	MALE	unknow	62	Stage IB	T2	M0	N0	NO	Lung Squamous Cell Carcinoma- Not Otherwise Specified (NOS)
TCGA-NK-A5CT	1992	0	MALE	WHITE	70	Stage IA	T1	M0	N0	NO	Lung Squamous Cell Carcinoma- Not Otherwise Specified (NOS)
TCGA-77-8007	198	1	MALE	unknow	68	Stage IIB	T2	M0	N1	unknow	Lung Squamous Cell Carcinoma- Not Otherwise Specified (NOS)
TCGA-56-A49D	637	0	MALE	WHITE	67	Stage IIIA	T2a	MX	N2	NO	Lung Squamous Cell Carcinoma- Not Otherwise Specified (NOS)
TCGA-66-2755	28	0	MALE	unknow	63	Stage IB	T2	M0	N0	unknow	Lung Squamous Cell Carcinoma- Not Otherwise Specified (NOS)
TCGA-43-8116	358	0	MALE	WHITE	73	Stage IA	T1b	M0	N0	NO	Lung Squamous Cell Carcinoma- Not Otherwise Specified (NOS)
TCGA-77-A5FZ	3838	1	MALE	unknow	64	Stage IIIB	T4	M0	N0	unknow	Lung Papillary Squamous Cell Caricnoma
TCGA-85-8049	579	0	MALE	WHITE	57	Stage IB	T2a	M0	N0	NO	Lung Squamous Cell Carcinoma- Not Otherwise Specified (NOS)
TCGA-85-8481	236	1	MALE	WHITE	70	Stage IIB	T3	M0	N0	unknow	Lung Papillary Squamous Cell Caricnoma
TCGA-43-6770	310	0	FEMALE	BLACK OR AFRICAN AMERICAN	59	Stage IB	T2a	MX	N0	unknow	Lung Squamous Cell Carcinoma- Not Otherwise Specified (NOS)
TCGA-56-8624	420	0	MALE	WHITE	84	Stage IIB	T3	MX	N0	NO	Lung Squamous Cell Carcinoma- Not Otherwise Specified (NOS)
TCGA-60-2722	908	0	MALE	WHITE	66	Stage IIB	T2	M0	N1	unknow	Lung Squamous Cell Carcinoma- Not Otherwise Specified (NOS)
TCGA-37-A5EN	660	0	MALE	WHITE	59	Stage IIIB	T4	M0	N2	NO	Lung Squamous Cell Carcinoma- Not Otherwise Specified (NOS)
TCGA-58-8386	1	1	MALE	unknow	75	Stage IV	T3	M1	NX	NO	Lung Squamous Cell Carcinoma- Not Otherwise Specified (NOS)
TCGA-60-2695	642	0	FEMALE	WHITE	74	Stage IB	T2	M0	N0	unknow	Lung Basaloid Squamous Cell Carcinoma
TCGA-46-3767	396	0	MALE	WHITE	76	Stage IA	T1a	M0	N0	unknow	Lung Squamous Cell Carcinoma- Not Otherwise Specified (NOS)
TCGA-43-6647	379	0	FEMALE	WHITE	69	Stage IIB	T2b	MX	N1	unknow	Lung Squamous Cell Carcinoma- Not Otherwise Specified (NOS)
TCGA-77-7142	1286	0	FEMALE	WHITE	59	Stage IB	T2	M0	N0	unknow	Lung Squamous Cell Carcinoma- Not Otherwise Specified (NOS)
TCGA-85-A4PA	741	0	MALE	WHITE	61	Stage IB	T2a	M0	N0	unknow	Lung Squamous Cell Carcinoma- Not Otherwise Specified (NOS)
TCGA-21-1077	1058	1	MALE	WHITE	64	Stage IIB	T2	M0	N1	unknow	Lung Squamous Cell Carcinoma- Not Otherwise Specified (NOS)
TCGA-22-0944	223	1	MALE	WHITE	61	Stage IB	T2	M0	N0	unknow	Lung Squamous Cell Carcinoma- Not Otherwise Specified (NOS)

TCGA-60-2725	816	0	MALE	WHITE	74	Stage IB	T2	M0	N0	unknow	Lung Squamous Cell Carcinoma- Not Otherwise Specified (NOS)
TCGA-70-6722	367	0	MALE	ASIAN	47	Stage IIIA	T3	M0	N1	unknow	Lung Squamous Cell Carcinoma- Not Otherwise Specified (NOS)
TCGA-66-2788	699	0	MALE	unknow	56	Stage IB	T2	M0	N0	unknow	Lung Squamous Cell Carcinoma- Not Otherwise Specified (NOS)
TCGA-18-3409	3747	0	MALE	WHITE	74	Stage IA	T1	M0	N0	unknow	Lung Basaloid Squamous Cell Carcinoma
TCGA-63-A5MM	456	1	FEMALE	unknow	69	Stage IIB	T2	M0	N1	YES	Lung Squamous Cell Carcinoma- Not Otherwise Specified (NOS)
TCGA-33-4547	2419	0	MALE	WHITE	68	Stage IB	T2	M0	N0	unknow	Lung Squamous Cell Carcinoma- Not Otherwise Specified (NOS)
TCGA-90-7766	289	0	FEMALE	WHITE	66	Stage IA	T1b	MX	N0	unknow	Lung Squamous Cell Carcinoma- Not Otherwise Specified (NOS)
TCGA-22-5479	1651	0	MALE	WHITE	64	Stage IB	T2	M0	N0	unknow	Lung Squamous Cell Carcinoma- Not Otherwise Specified (NOS)
TCGA-33-AASD	2249	1	MALE	BLACK OR AFRICAN AMERICAN	83	Stage IA	T1	MX	N0	YES	Lung Squamous Cell Carcinoma- Not Otherwise Specified (NOS)
TCGA-NC-A5HK	128	0	FEMALE	WHITE	58	Stage IIB	T3	M0	N0	NO	Lung Squamous Cell Carcinoma- Not Otherwise Specified (NOS)
TCGA-56-7221	608	0	MALE	WHITE	79	Stage IB	T2	M0	N0	unknow	Lung Squamous Cell Carcinoma- Not Otherwise Specified (NOS)
TCGA-46-6025	324	0	MALE	WHITE	71	Stage IIB	T2b	M0	N1	unknow	Lung Squamous Cell Carcinoma- Not Otherwise Specified (NOS)
TCGA-52-7809	166	1	MALE	WHITE	74	Stage IB	T2	M0	N0	unknow	Lung Basaloid Squamous Cell Carcinoma
TCGA-66-2765	61	0	MALE	unknow	64	Stage IB	T2	M0	N0	unknow	Lung Squamous Cell Carcinoma- Not Otherwise Specified (NOS)
TCGA-77-8144	833	0	MALE	unknow	70	Stage IB	T2	M0	N0	unknow	Lung Squamous Cell Carcinoma- Not Otherwise Specified (NOS)
TCGA-43-5670	549	0	MALE	WHITE	70	Stage IIA	T2b	M0	N0	unknow	Lung Squamous Cell Carcinoma- Not Otherwise Specified (NOS)
TCGA-77-8131	383	1	MALE	unknow	72	Stage IB	T2	M0	NX	unknow	Lung Squamous Cell Carcinoma- Not Otherwise Specified (NOS)
TCGA-21-A5DI	979	0	MALE	WHITE	77	Stage IA	T1b	M0	N0	NO	Lung Squamous Cell Carcinoma- Not Otherwise Specified (NOS)
TCGA-51-4080	12	1	MALE	BLACK OR AFRICAN AMERICAN	65	Stage IIIB	T4	unknow	N1	unknow	Lung Basaloid Squamous Cell Carcinoma
TCGA-37-A5EM	867	0	MALE	WHITE	49	Stage II	T2	M0	N0	NO	Lung Squamous Cell Carcinoma- Not Otherwise Specified (NOS)
TCGA-22-5489	1912	1	MALE	WHITE	64	Stage IA	T1b	M0	N0	unknow	Lung Squamous Cell Carcinoma- Not Otherwise Specified (NOS)
TCGA-77-A5G7	180	1	MALE	unknow	63	Stage IA	T1	M0	N0	unknow	Lung Squamous Cell Carcinoma- Not Otherwise Specified (NOS)
TCGA-60-2696	109	1	FEMALE	BLACK OR AFRICAN AMERICAN	76	Stage IIA	T2	M0	N0	unknow	Lung Squamous Cell Carcinoma- Not Otherwise Specified (NOS)

TCGA-85-A50Z	493	0	MALE	WHITE	57	Stage IIA	T2b	M0	N0	unknow	Lung Squamous Cell Carcinoma- Not Otherwise Specified (NOS)
TCGA-77-6842	899	1	MALE	WHITE	79	Stage IIB	T2	M0	N1	unknow	Lung Squamous Cell Carcinoma- Not Otherwise Specified (NOS)
TCGA-85-A4JB	539	0	MALE	WHITE	74	Stage IIB	T3	M0	N0	unknow	Lung Squamous Cell Carcinoma- Not Otherwise Specified (NOS)
TCGA-66-2771	578	0	MALE	unknow	60	Stage IIB	T2	M0	N1	unknow	Lung Squamous Cell Carcinoma- Not Otherwise Specified (NOS)
TCGA-63-A5MR	2716	0	FEMALE	unknow	70	Stage IB	T2	M0	N0	NO	Lung Squamous Cell Carcinoma- Not Otherwise Specified (NOS)
TCGA-68-7756	202	0	MALE	WHITE	84	Stage IIIA	T4	MX	N1	unknow	Lung Squamous Cell Carcinoma- Not Otherwise Specified (NOS)
TCGA-96-A4JL	504	0	FEMALE	ASIAN	78	Stage IIA	T2a	M0	N1	NO	Lung Squamous Cell Carcinoma- Not Otherwise Specified (NOS)
TCGA-85-8584	383	1	MALE	WHITE	71	Stage IIA	T2a	M0	N1	unknow	Lung Papillary Squamous Cell Caricnoma
TCGA-22-5472	1975	1	MALE	WHITE	67	Stage IB	T2a	M0	N0	unknow	Lung Squamous Cell Carcinoma- Not Otherwise Specified (NOS)
TCGA-18-5595	827	1	MALE	unknow	50	Stage IB	T2	M0	N0	unknow	Lung Squamous Cell Carcinoma- Not Otherwise Specified (NOS)
TCGA-85-8048	765	0	MALE	WHITE	62	Stage IA	T1	M0	N0	NO	Lung Squamous Cell Carcinoma- Not Otherwise Specified (NOS)
TCGA-60-2698	311	1	MALE	WHITE	62	Stage IIB	T2	M0	N1	unknow	Lung Squamous Cell Carcinoma- Not Otherwise Specified (NOS)
TCGA-85-8288	402	1	MALE	WHITE	70	Stage IIA	T1b	M0	N1	NO	Lung Squamous Cell Carcinoma- Not Otherwise Specified (NOS)
TCGA-34-5234	1715	0	FEMALE	WHITE	71	Stage IA	T1	M0	N0	unknow	Lung Squamous Cell Carcinoma- Not Otherwise Specified (NOS)
TCGA-46-3765	396	0	FEMALE	WHITE	59	Stage IA	T1	M0	N0	unknow	Lung Squamous Cell Carcinoma- Not Otherwise Specified (NOS)
TCGA-66-2795	122	0	MALE	unknow	68	Stage IIIB	T4	M0	N1	unknow	Lung Squamous Cell Carcinoma- Not Otherwise Specified (NOS)
TCGA-LA-A446	401	0	MALE	WHITE	68	Stage IA	T1b	MX	N0	NO	Lung Squamous Cell Carcinoma- Not Otherwise Specified (NOS)
TCGA-77-7463	1423	1	MALE	WHITE	75	Stage IB	T2	M0	N0	unknow	Lung Squamous Cell Carcinoma- Not Otherwise Specified (NOS)
TCGA-33-AASI	1344	1	FEMALE	BLACK OR AFRICAN AMERICAN	65	Stage IIB	T2	MX	N1	NO	Lung Squamous Cell Carcinoma- Not Otherwise Specified (NOS)
TCGA-22-5473	1933	1	MALE	WHITE	78	unknow	T3	M0	N0	unknow	Lung Squamous Cell Carcinoma- Not Otherwise Specified (NOS)
TCGA-68-7755	83	0	FEMALE	WHITE	60	Stage IIA	T1b	M0	N1	unknow	Lung Squamous Cell Carcinoma- Not Otherwise Specified (NOS)
TCGA-NC-A5HO	930	0	FEMALE	WHITE	70	Stage IIIA	T3	M0	N1	NO	Lung Squamous Cell Carcinoma- Not Otherwise Specified (NOS)
TCGA-18-3407	136	1	MALE	ASIAN	72	Stage IB	T2	M0	N0	unknow	Lung Squamous Cell Carcinoma- Not Otherwise Specified (NOS)
TCGA-37-4135	207	0	MALE	WHITE	68	Stage IB	T2a	M0	N0	unknow	Lung Squamous Cell Carcinoma- Not Otherwise Specified (NOS)

TCGA-34-2596	80	1	MALE	WHITE	70	Stage IIB	T2	M0	N1	unknow	Lung Squamous Cell Carcinoma- Not Otherwise Specified (NOS)
TCGA-96-7544	1517	0	MALE	WHITE	83	Stage IIB	T2	MX	N1	unknow	Lung Squamous Cell Carcinoma- Not Otherwise Specified (NOS)
TCGA-68-7757	211	0	MALE	WHITE	74	Stage IA	T1b	MX	N0	NO	Lung Squamous Cell Carcinoma- Not Otherwise Specified (NOS)
TCGA-85-8354	658	0	MALE	WHITE	53	Stage IB	T2a	M0	N0	unknow	Lung Squamous Cell Carcinoma- Not Otherwise Specified (NOS)
TCGA-66-2756	30	0	MALE	unknow	68	Stage IIIB	T4	M0	N0	unknow	Lung Squamous Cell Carcinoma- Not Otherwise Specified (NOS)
TCGA-39-5036	1084	0	MALE	WHITE	73	Stage IB	T2	M0	N0	unknow	Lung Squamous Cell Carcinoma- Not Otherwise Specified (NOS)
TCGA-77-A5G6	678	1	MALE	unknow	66	Stage IIIA	T2	M0	N2	YES	Lung Squamous Cell Carcinoma- Not Otherwise Specified (NOS)
TCGA-77-6845	708	1	MALE	WHITE	69	Stage IIB	T3	M0	N0	unknow	Lung Squamous Cell Carcinoma- Not Otherwise Specified (NOS)
TCGA-22-5485	916	1	FEMALE	WHITE	58	Stage IA	T1a	M0	N0	unknow	Lung Squamous Cell Carcinoma- Not Otherwise Specified (NOS)
TCGA-90-6837	758	0	MALE	WHITE	64	Stage IIB	T3	MX	N0	unknow	Lung Squamous Cell Carcinoma- Not Otherwise Specified (NOS)
TCGA-33-4566	5287	1	MALE	WHITE	40	Stage IB	T2	M0	N0	unknow	Lung Squamous Cell Carcinoma- Not Otherwise Specified (NOS)
TCGA-46-3766	365	0	FEMALE	WHITE	62	Stage IA	T1	M0	N0	unknow	Lung Squamous Cell Carcinoma- Not Otherwise Specified (NOS)
TCGA-66-2777	61	0	MALE	unknow	71	Stage IB	T2	M0	N0	unknow	Lung Squamous Cell Carcinoma- Not Otherwise Specified (NOS)
TCGA-77-8143	803	1	MALE	unknow	76	Stage IIIA	T2	M0	N2	unknow	Lung Squamous Cell Carcinoma- Not Otherwise Specified (NOS)
TCGA-90-A4EE	688	0	MALE	WHITE	53	Stage IIA	T2a	MX	N1	NO	Lung Squamous Cell Carcinoma- Not Otherwise Specified (NOS)
TCGA-66-2785	60	0	MALE	unknow	65	Stage IB	T2	M0	N0	unknow	Lung Squamous Cell Carcinoma- Not Otherwise Specified (NOS)
TCGA-66-2789	123	1	MALE	unknow	73	Stage IIIB	T1	M0	N3	unknow	Lung Squamous Cell Carcinoma- Not Otherwise Specified (NOS)
TCGA-NC-A5HJ	418	1	MALE	WHITE	59	Stage IIB	T3	M0	N0	YES	Lung Squamous Cell Carcinoma- Not Otherwise Specified (NOS)
TCGA-46-3768	299	1	MALE	WHITE	58	Stage IIIA	T3	M0	N1	unknow	Lung Squamous Cell Carcinoma- Not Otherwise Specified (NOS)
TCGA-34-7107	34	1	MALE	WHITE	70	Stage II	T2a	M0	N0	unknow	Lung Squamous Cell Carcinoma- Not Otherwise Specified (NOS)
TCGA-77-8009	1641	0	MALE	unknow	68	Stage IIB	T2	M0	N1	unknow	Lung Squamous Cell Carcinoma- Not Otherwise Specified (NOS)
TCGA-98-A539	173	0	MALE	WHITE	63	Stage IIB	T3	M0	N0	NO	Lung Squamous Cell Carcinoma- Not Otherwise Specified (NOS)
TCGA-63-A5MS	2381	0	MALE	unknow	78	Stage IB	T2	M0	N0	NO	Lung Squamous Cell Carcinoma- Not Otherwise Specified (NOS)
TCGA-60-2714	512	0	FEMALE	WHITE	66	Stage IIB	T2	M0	N1	unknow	Lung Squamous Cell Carcinoma- Not Otherwise Specified (NOS)

TCGA-33-4533	4068	0	FEMALE	BLACK OR AFRICAN AMERICAN	76	Stage IB	T2	M0	N0	unknow	Lung Squamous Cell Carcinoma- Not Otherwise Specified (NOS)
TCGA-18-3412	345	1	MALE	WHITE	52	Stage IB	T2	M0	N0	unknow	Lung Squamous Cell Carcinoma- Not Otherwise Specified (NOS)
TCGA-43-3394	433	0	MALE	BLACK OR AFRICAN AMERICAN	52	Stage IB	T2a	M0	N0	unknow	Lung Squamous Cell Carcinoma- Not Otherwise Specified (NOS)
TCGA-33-4586	428	1	MALE	WHITE	57	Stage IIIA	T2	M0	N2	unknow	Lung Squamous Cell Carcinoma- Not Otherwise Specified (NOS)
TCGA-92-7340	82	0	FEMALE	WHITE	45	Stage IIA	T2a	MX	N1	unknow	Lung Squamous Cell Carcinoma- Not Otherwise Specified (NOS)
TCGA-58-A46L	1723	0	MALE	WHITE	73	Stage IIIA	T2	M0	N2	NO	Lung Basaloid Squamous Cell Carcinoma
TCGA-85-7697	1063	0	MALE	WHITE	49	Stage IIB	T3	M0	N0	unknow	Lung Squamous Cell Carcinoma- Not Otherwise Specified (NOS)
TCGA-85-A4CL	781	0	MALE	WHITE	65	Stage IA	T1b	M0	N0	unknow	Lung Squamous Cell Carcinoma- Not Otherwise Specified (NOS)
TCGA-60-2706	2500	0	MALE	WHITE	58	Stage IA	T1	M0	N0	unknow	Lung Squamous Cell Carcinoma- Not Otherwise Specified (NOS)
TCGA-22-0940	669	1	MALE	WHITE	71	Stage IIA	T1	M0	N1	unknow	Lung Squamous Cell Carcinoma- Not Otherwise Specified (NOS)
TCGA-56-7823	1011	0	FEMALE	WHITE	58	Stage IIA	T1b	M0	N1	NO	Lung Squamous Cell Carcinoma- Not Otherwise Specified (NOS)
TCGA-98-A53D	645	1	MALE	WHITE	68	Stage IIB	T3	M0	N0	NO	Lung Squamous Cell Carcinoma- Not Otherwise Specified (NOS)
TCGA-77-7337	3253	1	MALE	WHITE	65	Stage IIB	T2	M0	N1	unknow	Lung Squamous Cell Carcinoma- Not Otherwise Specified (NOS)
TCGA-51-4081	63	0	MALE	WHITE	55	Stage IIB	T2a	M0	N1	unknow	Lung Squamous Cell Carcinoma- Not Otherwise Specified (NOS)
TCGA-98-8023	649	0	MALE	WHITE	70	Stage IIIA	T3	M0	N1	NO	Lung Squamous Cell Carcinoma- Not Otherwise Specified (NOS)
TCGA-34-2605	881	1	MALE	WHITE	76	Stage IIIB	T4	M0	N1	unknow	Lung Squamous Cell Carcinoma- Not Otherwise Specified (NOS)
TCGA-56-1622	881	1	MALE	WHITE	58	Stage IB	T2	M0	N0	unknow	Lung Squamous Cell Carcinoma- Not Otherwise Specified (NOS)
TCGA-77-8139	3166	0	MALE	unknow	72	Stage IIB	T3	M0	N0	NO	Lung Squamous Cell Carcinoma- Not Otherwise Specified (NOS)
TCGA-66-2758	639	0	MALE	unknow	71	Stage IB	T2	M0	N0	unknow	Lung Squamous Cell Carcinoma- Not Otherwise Specified (NOS)
TCGA-NC-A5HT	693	0	MALE	WHITE	69	Stage IIIA	T3	M0	N1	YES	Lung Squamous Cell Carcinoma- Not Otherwise Specified (NOS)
TCGA-56-8622	55	0	MALE	WHITE	68	Stage IB	T2a	M0	N0	NO	Lung Squamous Cell Carcinoma- Not Otherwise Specified (NOS)
TCGA-22-1000	454	1	MALE	WHITE	76	Stage IB	T2	M0	N0	unknow	Lung Squamous Cell Carcinoma- Not Otherwise Specified (NOS)
TCGA-NK-A5CR	2542	0	MALE	unknow	77	Stage IB	T2	MX	N0	NO	Lung Squamous Cell Carcinoma- Not Otherwise Specified (NOS)
TCGA-56-8623	692	1	MALE	WHITE	71	Stage IB	T2a	MX	N0	NO	Lung Squamous Cell Carcinoma- Not Otherwise Specified (NOS)

TCGA-92-7341	106	0	MALE	WHITE	71	Stage IB	T2a	MX	N0	unknow	Lung Squamous Cell Carcinoma- Not Otherwise Specified (NOS)
TCGA-56-8629	481	0	MALE	WHITE	63	Stage IIA	T2b	MX	N0	NO	Lung Squamous Cell Carcinoma- Not Otherwise Specified (NOS)
TCGA-22-1002	131	1	MALE	WHITE	69	Stage IA	T1	M0	N0	unknow	Lung Squamous Cell Carcinoma- Not Otherwise Specified (NOS)
TCGA-77-6844	2284	1	MALE	WHITE	74	Stage IIIA	T3	M0	N1	unknow	Lung Squamous Cell Carcinoma- Not Otherwise Specified (NOS)
TCGA-21-1076	1852	0	FEMALE	WHITE	54	Stage IB	T2	M0	N0	unknow	Lung Squamous Cell Carcinoma- Not Otherwise Specified (NOS)
TCGA-56-8504	510	0	MALE	WHITE	74	Stage IB	T2a	MX	N0	NO	Lung Squamous Cell Carcinoma- Not Otherwise Specified (NOS)
TCGA-56-7731	3	1	FEMALE	WHITE	66	Stage IB	T2a	MX	N0	unknow	Lung Squamous Cell Carcinoma- Not Otherwise Specified (NOS)
TCGA-77-6843	2224	1	MALE	WHITE	74	Stage IIA	T1	M0	N1	unknow	Lung Squamous Cell Carcinoma- Not Otherwise Specified (NOS)
TCGA-21-5782	962	1	FEMALE	WHITE	68	Stage IB	T2	M0	N0	unknow	Lung Squamous Cell Carcinoma- Not Otherwise Specified (NOS)
TCGA-90-A4ED	615	0	MALE	WHITE	69	Stage IB	T2a	MX	N0	NO	Lung Squamous Cell Carcinoma- Not Otherwise Specified (NOS)
TCGA-43-2576	556	0	FEMALE	WHITE	62	Stage IIIA	T2	M0	N2	unknow	Lung Squamous Cell Carcinoma- Not Otherwise Specified (NOS)
TCGA-18-3417	1097	1	MALE	unknow	65	Stage IV	T2	M1	N1	unknow	Lung Squamous Cell Carcinoma- Not Otherwise Specified (NOS)
TCGA-66-2800	1492	0	MALE	unknow	70	Stage IIIB	T4	M0	N0	unknow	Lung Squamous Cell Carcinoma- Not Otherwise Specified (NOS)
TCGA-21-1070	3636	0	FEMALE	BLACK OR AFRICAN AMERICAN	60	Stage IIIA	T3	M0	N0	unknow	Lung Squamous Cell Carcinoma- Not Otherwise Specified (NOS)
TCGA-63-A5MV	1100	0	MALE	unknow	69	Stage IIA	T2b	M0	N0	NO	Lung Squamous Cell Carcinoma- Not Otherwise Specified (NOS)
TCGA-66-2793	306	1	MALE	unknow	68	Stage IIIB	T4	M0	N1	unknow	Lung Squamous Cell Carcinoma- Not Otherwise Specified (NOS)
TCGA-33-4587	1656	1	FEMALE	WHITE	63	Stage IB	T2	MX	N0	unknow	Lung Small Cell Squamous Cell Carcinoma
TCGA-33-6737	601	1	MALE	WHITE	71	Stage IIIA	T2	M0	N2	unknow	Lung Squamous Cell Carcinoma- Not Otherwise Specified (NOS)
TCGA-77-8136	1189	1	FEMALE	unknow	74	Stage IIB	T2	M0	N1	YES	Lung Squamous Cell Carcinoma- Not Otherwise Specified (NOS)
TCGA-85-7699	1001	1	MALE	WHITE	73	Stage IIIA	T4	M0	N0	unknow	Lung Squamous Cell Carcinoma- Not Otherwise Specified (NOS)
TCGA-66-2727	516	1	FEMALE	unknow	55	Stage IB	T2	M0	N0	unknow	Lung Squamous Cell Carcinoma- Not Otherwise Specified (NOS)
TCGA-85-A513	461	0	FEMALE	ASIAN	60	Stage IA	T1a	M0	NX	unknow	Lung Squamous Cell Carcinoma- Not Otherwise Specified (NOS)
TCGA-85-8479	468	0	MALE	WHITE	66	Stage IA	T1a	M0	N0	unknow	Lung Papillary Squamous Cell Caricnoma
TCGA-22-4609	291	1	MALE	WHITE	81	Stage IA	T1	M0	N0	unknow	Lung Squamous Cell Carcinoma- Not Otherwise Specified (NOS)

TCGA-39-5027	1849	0	MALE	WHITE	73	Stage IB	T2a	M0	N0	unknow	Lung Squamous Cell Carcinoma- Not Otherwise Specified (NOS)
TCGA-92-8063	122	0	MALE	WHITE	52	Stage IIIA	T2b	MX	N2	NO	Lung Squamous Cell Carcinoma- Not Otherwise Specified (NOS)
TCGA-60-2723	693	0	FEMALE	WHITE	74	Stage IB	T2	M0	N0	unknow	Lung Squamous Cell Carcinoma- Not Otherwise Specified (NOS)
TCGA-34-5236	276	1	MALE	WHITE	60	Stage IIB	T3	M0	N0	unknow	Lung Squamous Cell Carcinoma- Not Otherwise Specified (NOS)
TCGA-37-3789	13	0	MALE	WHITE	65	Stage IB	T2	unknow	N0	unknow	Lung Squamous Cell Carcinoma- Not Otherwise Specified (NOS)
TCGA-63-A5MG	2148	0	MALE	unknow	68	Stage IB	T2	M0	N0	NO	Lung Squamous Cell Carcinoma- Not Otherwise Specified (NOS)
TCGA-33-A4WN	143	1	MALE	WHITE	60	Stage IB	T2a	MX	N0	NO	Lung Squamous Cell Carcinoma- Not Otherwise Specified (NOS)
TCGA-94-A5I6	538	0	MALE	WHITE	62	Stage IIB	T3	M0	N0	NO	Lung Squamous Cell Carcinoma- Not Otherwise Specified (NOS)
TCGA-85-8664	434	0	MALE	WHITE	73	Stage IIB	T2b	M0	N1	unknow	Lung Squamous Cell Carcinoma- Not Otherwise Specified (NOS)
TCGA-22-4596	17	1	FEMALE	WHITE	69	Stage IB	T1b	M0	N0	unknow	Lung Squamous Cell Carcinoma- Not Otherwise Specified (NOS)
TCGA-39-5029	740	1	MALE	WHITE	67	Stage IIIA	T1b	M0	N2	unknow	Lung Squamous Cell Carcinoma- Not Otherwise Specified (NOS)
TCGA-43-2581	399	0	FEMALE	WHITE	47	Stage IIIA	T3	M0	N1	unknow	Lung Squamous Cell Carcinoma- Not Otherwise Specified (NOS)
TCGA-94-A4VJ	430	0	FEMALE	WHITE	71	Stage IA	T1b	M0	N0	NO	Lung Squamous Cell Carcinoma- Not Otherwise Specified (NOS)
TCGA-NC-A5HN	1132	0	MALE	WHITE	77	Stage IIA	T2a	M0	N1	NO	Lung Squamous Cell Carcinoma- Not Otherwise Specified (NOS)
TCGA-39-5037	1086	0	MALE	WHITE	65	Stage IIA	T1b	M0	N1	unknow	Lung Squamous Cell Carcinoma- Not Otherwise Specified (NOS)
TCGA-60-2726	358	1	MALE	WHITE	56	Stage IIA	T2	M0	N1	unknow	Lung Squamous Cell Carcinoma- Not Otherwise Specified (NOS)
TCGA-98-A53J	630	0	MALE	WHITE	77	Stage IB	T2a	M0	N0	NO	Lung Squamous Cell Carcinoma- Not Otherwise Specified (NOS)
TCGA-66-2737	61	0	MALE	unknow	72	Stage IIB	T2	M0	N1	unknow	Lung Squamous Cell Carcinoma- Not Otherwise Specified (NOS)
TCGA-56-A4ZK	570	0	FEMALE	WHITE	76	Stage IB	T2	M0	N0	NO	Lung Squamous Cell Carcinoma- Not Otherwise Specified (NOS)
TCGA-77-A5GA	1280	0	MALE	unknow	76	Stage IB	T2a	M0	N0	unknow	Lung Squamous Cell Carcinoma- Not Otherwise Specified (NOS)
TCGA-60-2703	2945	1	MALE	WHITE	73	Stage IIB	T2	M0	N1	unknow	Lung Squamous Cell Carcinoma- Not Otherwise Specified (NOS)
TCGA-22-5481	2409	1	FEMALE	WHITE	72	Stage IIB	T2	M0	N1	unknow	Lung Squamous Cell Carcinoma- Not Otherwise Specified (NOS)
TCGA-22-4613	358	1	FEMALE	WHITE	73	Stage IA	T1b	M0	N0	unknow	Lung Squamous Cell Carcinoma- Not Otherwise Specified (NOS)
TCGA-56-A5DR	4	0	MALE	WHITE	81	Stage IA	T1a	MX	N0	NO	Lung Squamous Cell Carcinoma- Not Otherwise Specified (NOS)
TCGA-77-7141	15	0	MALE	WHITE	64	Stage IB	T2	M0	N0	unknow	Lung Squamous Cell Carcinoma- Not Otherwise Specified (NOS)

TCGA-58-A46N	910	0	MALE	WHITE	52	Stage IB	T2a	M0	N0	NO	Lung Basaloid Squamous Cell Carcinoma
TCGA-63-A5MP	769	0	MALE	unknow	56	Stage IIB	T2	M0	N1	YES	Lung Squamous Cell Carcinoma- Not Otherwise Specified (NOS)
TCGA-43-7658	2023	0	FEMALE	WHITE	75	Stage IA	T1	M0	N0	unknow	Lung Squamous Cell Carcinoma- Not Otherwise Specified (NOS)
TCGA-43-A56U	432	0	FEMALE	WHITE	76	Stage IA	T1b	MX	N0	NO	Lung Squamous Cell Carcinoma- Not Otherwise Specified (NOS)
TCGA-L3-A4E7	392	0	MALE	WHITE	71	Stage IB	T2a	M0	N0	NO	Lung Squamous Cell Carcinoma- Not Otherwise Specified (NOS)
TCGA-56-7730	198	1	MALE	WHITE	73	Stage IIA	T2b	M0	N0	unknow	Lung Squamous Cell Carcinoma- Not Otherwise Specified (NOS)
TCGA-22-4605	974	1	FEMALE	unknow	78	Stage IB	T2	M0	N0	unknow	Lung Squamous Cell Carcinoma- Not Otherwise Specified (NOS)
TCGA-56-8626	302	1	MALE	WHITE	59	Stage IA	T1a	MX	N0	NO	Lung Squamous Cell Carcinoma- Not Otherwise Specified (NOS)
TCGA-85-8355	61	0	MALE	WHITE	63	Stage IA	T1a	M0	N0	unknow	Lung Squamous Cell Carcinoma- Not Otherwise Specified (NOS)
TCGA-90-7769	358	0	MALE	WHITE	55	Stage IIB	T2b	MX	N1	unknow	Lung Squamous Cell Carcinoma- Not Otherwise Specified (NOS)
TCGA-43-6771	166	1	MALE	WHITE	85	Stage IB	T2	MX	N0	unknow	Lung Squamous Cell Carcinoma- Not Otherwise Specified (NOS)
TCGA-63-7021		0	MALE	unknow	unknow	Stage IA	T1	M0	N0	unknow	Lung Squamous Cell Carcinoma- Not Otherwise Specified (NOS)
TCGA-34-5928	552	0	FEMALE	WHITE	83	Stage IIB	T2	M0	N1	unknow	Lung Squamous Cell Carcinoma- Not Otherwise Specified (NOS)
TCGA-60-2704	1154	1	MALE	WHITE	73	Stage IIB	T2	M0	N1	unknow	Lung Squamous Cell Carcinoma- Not Otherwise Specified (NOS)
TCGA-56-8308	517	0	MALE	WHITE	79	Stage IIB	T3	MX	N0	NO	Lung Squamous Cell Carcinoma- Not Otherwise Specified (NOS)
TCGA-85-8070	510	0	MALE	WHITE	71	Stage IB	T2	M0	N0	unknow	Lung Squamous Cell Carcinoma- Not Otherwise Specified (NOS)
TCGA-NC-A5HM	1212	0	MALE	WHITE	76	Stage IB	T2a	M0	N0	NO	Lung Squamous Cell Carcinoma- Not Otherwise Specified (NOS)
TCGA-33-6738	822	0	MALE	WHITE	80	Stage IIIA	T1	MX	N2	unknow	Lung Squamous Cell Carcinoma- Not Otherwise Specified (NOS)
TCGA-43-A475	296	0	FEMALE	WHITE	67	Stage IIB	T3	M0	N0	NO	Lung Squamous Cell Carcinoma- Not Otherwise Specified (NOS)
TCGA-NC-A5HF	138	1	MALE	WHITE	74	Stage IIIB	T4	MX	N0	YES	Lung Squamous Cell Carcinoma- Not Otherwise Specified (NOS)
TCGA-NC-A5HP	730	0	MALE	WHITE	69	Stage IV	T2a	M1b	N0	YES	Lung Squamous Cell Carcinoma- Not Otherwise Specified (NOS)
TCGA-94-A5I4	491	0	MALE	WHITE	61	Stage IIA	T2a	MX	N1	NO	Lung Squamous Cell Carcinoma- Not Otherwise Specified (NOS)
TCGA-90-7767	89	0	MALE	WHITE	56	Stage IIB	T2b	MX	N1	unknow	Lung Squamous Cell Carcinoma- Not Otherwise Specified (NOS)
TCGA-66-2792	913	0	MALE	unknow	58	Stage IIB	T2	M0	N1	unknow	Lung Squamous Cell Carcinoma- Not Otherwise Specified (NOS)

TCGA-77-8133	1640	1	MALE	unknow	74	Stage IIA	T1	M0	N1	unknow	Lung Squamous Cell Carcinoma- Not Otherwise Specified (NOS)
TCGA-18-3411	3576	0	FEMALE	unknow	63	Stage IIIA	T2	M0	N2	unknow	Lung Squamous Cell Carcinoma- Not Otherwise Specified (NOS)
TCGA-33-A5GW	9	0	MALE	WHITE	67	Stage IIA	T1a	MX	N1	NO	Lung Squamous Cell Carcinoma- Not Otherwise Specified (NOS)
TCGA-18-4086	85	1	MALE	unknow	64	Stage IB	T2	M0	N0	unknow	Lung Squamous Cell Carcinoma- Not Otherwise Specified (NOS)
TCGA-43-A474	353	0	MALE	WHITE	66	Stage IIA	T2b	M0	N0	NO	Lung Squamous Cell Carcinoma- Not Otherwise Specified (NOS)
TCGA-XC-AA0X	6	1	FEMALE	BLACK OR AFRICAN AMERICAN	77	Stage IA	T1a	M0	N0	NO	Lung Squamous Cell Carcinoma- Not Otherwise Specified (NOS)
TCGA-34-8455	123	1	MALE	WHITE	67	Stage IV	T4	M1a	N0	NO	Lung Squamous Cell Carcinoma- Not Otherwise Specified (NOS)
TCGA-33-4532	2524	0	MALE	WHITE	68	Stage IB	T2	M0	N0	unknow	Lung Squamous Cell Carcinoma- Not Otherwise Specified (NOS)
TCGA-39-5011	1454	0	FEMALE	WHITE	70	Stage IA	T1b	M0	N0	unknow	Lung Squamous Cell Carcinoma- Not Otherwise Specified (NOS)
TCGA-56-A62T	84	0	MALE	BLACK OR AFRICAN AMERICAN	78	Stage IIA	T2b	MX	N0	NO	Lung Squamous Cell Carcinoma- Not Otherwise Specified (NOS)
TCGA-37-4130	247	0	MALE	WHITE	56	Stage IA	T1b	M0	N0	unknow	Lung Squamous Cell Carcinoma- Not Otherwise Specified (NOS)
TCGA-96-8169	557	0	FEMALE	WHITE	67	Stage IA	T1a	M0	N0	NO	Lung Squamous Cell Carcinoma- Not Otherwise Specified (NOS)
TCGA-34-5927	941	0	FEMALE	WHITE	70	Stage IA	T1	M0	N0	unknow	Lung Squamous Cell Carcinoma- Not Otherwise Specified (NOS)
TCGA-22-5482	357	1	MALE	WHITE	81	Stage IB	T2a	M0	N0	unknow	Lung Squamous Cell Carcinoma- Not Otherwise Specified (NOS)
TCGA-85-A4JC	455	0	MALE	WHITE	84	Stage IIA	T2b	M0	N0	unknow	Lung Squamous Cell Carcinoma- Not Otherwise Specified (NOS)
TCGA-85-A50M	382	0	MALE	ASIAN	47	Stage IIA	T2b	M0	N0	NO	Lung Squamous Cell Carcinoma- Not Otherwise Specified (NOS)
TCGA-22-A5C4	671	0	MALE	WHITE	70	Stage IIA	T2b	M0	N0	NO	Lung Squamous Cell Carcinoma- Not Otherwise Specified (NOS)
TCGA-85-8276	1050	0	MALE	WHITE	62	Stage IIA	T1b	M0	N1	NO	Lung Squamous Cell Carcinoma- Not Otherwise Specified (NOS)
TCGA-60-2711	1014	0	FEMALE	WHITE	64	Stage IB	T2	M0	N0	unknow	Lung Squamous Cell Carcinoma- Not Otherwise Specified (NOS)
TCGA-43-3920	357	0	MALE	WHITE	71	Stage IB	T2	M0	N0	unknow	Lung Squamous Cell Carcinoma- Not Otherwise Specified (NOS)
TCGA-56-8503	41	0	FEMALE	WHITE	76	Stage IIB	T3	M0	N0	NO	Lung Squamous Cell Carcinoma- Not Otherwise Specified (NOS)
TCGA-56-A4BW	585	0	MALE	WHITE	55	Stage IIA	T2a	M0	N1	NO	Lung Squamous Cell Carcinoma- Not Otherwise Specified (NOS)
TCGA-33-4538	2979	1	MALE	WHITE	66	Stage IIIA	T2	M0	N2	unknow	Lung Squamous Cell Carcinoma- Not Otherwise Specified (NOS)
TCGA-39-5022	1679	1	MALE	WHITE	76	Stage IB	T2a	M0	N0	unknow	Lung Squamous Cell Carcinoma- Not Otherwise Specified (NOS)

TCGA-98-8020	84	1	FEMALE	BLACK OR AFRICAN AMERICAN	56	Stage IIIA	T2	M0	N2	YES	Lung Squamous Cell Carcinoma- Not Otherwise Specified (NOS)
TCGA-22-4594	1470	1	FEMALE	unknow	60	Stage IIIA	T3	M0	N2	unknow	Lung Squamous Cell Carcinoma- Not Otherwise Specified (NOS)
TCGA-33-AASB	211	1	MALE	BLACK OR AFRICAN AMERICAN	66	Stage IB	T2	MX	N0	YES	Lung Squamous Cell Carcinoma- Not Otherwise Specified (NOS)
TCGA-37-3783	122	0	MALE	WHITE	51	Stage IIIA	T3	M0	N2	unknow	Lung Squamous Cell Carcinoma- Not Otherwise Specified (NOS)
TCGA-92-8064	160	0	MALE	WHITE	58	unknow	T2b	MX	N0	NO	Lung Squamous Cell Carcinoma- Not Otherwise Specified (NOS)
TCGA-NC-A5HI	1380	0	FEMALE	WHITE	68	Stage IB	T2	M0	N0	YES	Lung Squamous Cell Carcinoma- Not Otherwise Specified (NOS)
TCGA-NC-A5HD	2	1	MALE	WHITE	79	Stage IIB	T3	M0	N0	NO	Lung Squamous Cell Carcinoma- Not Otherwise Specified (NOS)
TCGA-39-5030	59	1	FEMALE	ASIAN	81	Stage IIIA	T2a	M0	N2	unknow	Lung Squamous Cell Carcinoma- Not Otherwise Specified (NOS)
TCGA-98-A53I	565	0	MALE	WHITE	64	Stage IIA	T2a	M0	N1	NO	Lung Squamous Cell Carcinoma- Not Otherwise Specified (NOS)
TCGA-43-7657	236	0	FEMALE	WHITE	68	Stage IA	T1	MX	N0	unknow	Lung Squamous Cell Carcinoma- Not Otherwise Specified (NOS)
TCGA-77-8148	293	0	MALE	unknow	68	Stage IIIA	T3	M0	N1	unknow	Lung Squamous Cell Carcinoma- Not Otherwise Specified (NOS)
TCGA-66-2767	61	0	MALE	unknow	62	Stage IIIB	T2	M0	N3	unknow	Lung Squamous Cell Carcinoma- Not Otherwise Specified (NOS)
TCGA-85-A4CN	612	0	FEMALE	WHITE	56	Stage IIB	T2b	M0	N1	NO	Lung Squamous Cell Carcinoma- Not Otherwise Specified (NOS)
TCGA-77-7139	2737	0	MALE	WHITE	56	Stage IIB	T2	M0	N1	unknow	Lung Squamous Cell Carcinoma- Not Otherwise Specified (NOS)
TCGA-02-A52W	261	1	MALE	BLACK OR AFRICAN AMERICAN	63	Stage I	T2	MX	N0	NO	Lung Squamous Cell Carcinoma- Not Otherwise Specified (NOS)
TCGA-63-A5MW	1639	0	MALE	unknow	76	Stage IB	T2	M0	N0	NO	Lung Squamous Cell Carcinoma- Not Otherwise Specified (NOS)
TCGA-66-2770	700	0	MALE	unknow	79	Stage IB	T2	M0	N0	unknow	Lung Squamous Cell Carcinoma- Not Otherwise Specified (NOS)
TCGA-66-2754	61	0	MALE	unknow	67	Stage IIIA	T2	M0	N2	unknow	Lung Squamous Cell Carcinoma- Not Otherwise Specified (NOS)
TCGA-18-3410	146	1	MALE	unknow	81	Stage IIB	T3	M0	N0	unknow	Lung Squamous Cell Carcinoma- Not Otherwise Specified (NOS)
TCGA-77-7338	5	1	MALE	WHITE	64	Stage IB	T2	M0	N0	unknow	Lung Squamous Cell Carcinoma- Not Otherwise Specified (NOS)
TCGA-85-7844	911	0	MALE	WHITE	71	Stage IB	T2a	M0	N0	unknow	Lung Squamous Cell Carcinoma- Not Otherwise Specified (NOS)
TCGA-92-8065	70	0	FEMALE	WHITE	74	Stage IIB	T3	MX	N0	NO	Lung Squamous Cell Carcinoma- Not Otherwise Specified (NOS)
TCGA-NC-A5HQ	448	1	MALE	WHITE	70	Stage IIIA	T3	M0	N2	NO	Lung Squamous Cell Carcinoma- Not Otherwise Specified (NOS)

TCGA-60-2720	97	0	FEMALE	WHITE	60	Stage IB	T2	M0	N0	unknow	Lung Squamous Cell Carcinoma- Not Otherwise Specified (NOS)
TCGA-22-1017	1485	1	MALE	WHITE	62	Stage IA	T1	M0	N0	unknow	Lung Squamous Cell Carcinoma- Not Otherwise Specified (NOS)
TCGA-21-1082	3644	0	MALE	WHITE	61	Stage IB	T2	M0	N0	unknow	Lung Squamous Cell Carcinoma- Not Otherwise Specified (NOS)
TCGA-22-5477	1346	1	MALE	WHITE	65	Stage IA	T1	M0	N0	unknow	Lung Squamous Cell Carcinoma- Not Otherwise Specified (NOS)
TCGA-56-7223	442	1	MALE	WHITE	66	Stage IIIA	T3	MX	N1	unknow	Lung Squamous Cell Carcinoma- Not Otherwise Specified (NOS)
TCGA-43-8115	407	0	FEMALE	WHITE	72	Stage IIA	T2a	MX	N1	NO	Lung Squamous Cell Carcinoma- Not Otherwise Specified (NOS)
TCGA-85-A5B5	111	0	MALE	WHITE	58	Stage IA	T1b	M0	N0	NO	Lung Squamous Cell Carcinoma- Not Otherwise Specified (NOS)
TCGA-63-A5M9	0	0	FEMALE	unknow	unk now	Stage IIB	T2	M0	N1	unknow	Lung Squamous Cell Carcinoma- Not Otherwise Specified (NOS)
TCGA-56-5897	378	0	MALE	WHITE	74	Stage IA	T1b	MX	N0	unknow	Lung Squamous Cell Carcinoma- Not Otherwise Specified (NOS)
TCGA-77-7465	479	0	MALE	WHITE	58	Stage IIA	T2a	M0	N1	unknow	Lung Squamous Cell Carcinoma- Not Otherwise Specified (NOS)
TCGA-L3-A524	490	1	FEMALE	WHITE	45	Stage IIB	T3	M0	N0	NO	Lung Squamous Cell Carcinoma- Not Otherwise Specified (NOS)
TCGA-22-4601	1057	1	FEMALE	WHITE	73	Stage IIIA	T4	M0	N0	unknow	Lung Squamous Cell Carcinoma- Not Otherwise Specified (NOS)
TCGA-58-8393	1058	0	FEMALE	WHITE	68	Stage IB	T2a	M0	N0	NO	Lung Squamous Cell Carcinoma- Not Otherwise Specified (NOS)
TCGA-68-8251	406	0	MALE	WHITE	78	Stage IB	T2a	M0	N0	NO	Lung Squamous Cell Carcinoma- Not Otherwise Specified (NOS)
TCGA-63-5131		1	MALE	unknow	unk now	Stage IIB	T2	M0	N1	unknow	Lung Squamous Cell Carcinoma- Not Otherwise Specified (NOS)
TCGA-22-4604	399	1	MALE	WHITE	73	Stage IIA	T2a	M0	N1	unknow	Lung Squamous Cell Carcinoma- Not Otherwise Specified (NOS)
TCGA-56-7579	951	1	MALE	WHITE	61	Stage IIIA	T3	M0	N1	unknow	Lung Squamous Cell Carcinoma- Not Otherwise Specified (NOS)
TCGA-37-4132	227	0	FEMALE	WHITE	61	Stage IV	T2	M1	N0	unknow	Lung Squamous Cell Carcinoma- Not Otherwise Specified (NOS)
TCGA-56-7580	925	0	MALE	WHITE	84	Stage IB	T2a	M0	N0	unknow	Lung Squamous Cell Carcinoma- Not Otherwise Specified (NOS)
TCGA-77-A5GB	0	1	MALE	unknow	90	Stage IB	T2a	M0	NX	YES	Lung Squamous Cell Carcinoma- Not Otherwise Specified (NOS)
TCGA-58-8391	2167	0	FEMALE	WHITE	57	Stage IIIA	T2	M0	N2	NO	Lung Squamous Cell Carcinoma- Not Otherwise Specified (NOS)
TCGA-68-8250	244	0	MALE	BLACK OR AFRICAN AMERICAN	66	Stage IA	T1a	MX	N0	NO	Lung Squamous Cell Carcinoma- Not Otherwise Specified (NOS)
TCGA-39-5024	2130	0	FEMALE	WHITE	65	Stage IIIA	T2a	M0	N2	unknow	Lung Squamous Cell Carcinoma- Not Otherwise Specified (NOS)

TCGA-21-1071	1426	1	MALE	WHITE	67	Stage IB	T2	M0	N0	unknow	Lung Squamous Cell Carcinoma- Not Otherwise Specified (NOS)
TCGA-56-6545	666	0	FEMALE	WHITE	77	Stage IB	T2a	M0	N0	unknow	Lung Squamous Cell Carcinoma- Not Otherwise Specified (NOS)
TCGA-96-A4JK	589	0	MALE	WHITE	65	Stage IIA	T2a	M0	N1	NO	Lung Squamous Cell Carcinoma- Not Otherwise Specified (NOS)
TCGA-22-1011	53	1	MALE	WHITE	73	Stage IB	T2	M0	N0	unknow	Lung Squamous Cell Carcinoma- Not Otherwise Specified (NOS)
TCGA-21-5784	908	0	FEMALE	WHITE	80	Stage IB	T2	M0	N0	unknow	Lung Squamous Cell Carcinoma- Not Otherwise Specified (NOS)
TCGA-43-6143	376	0	MALE	WHITE	70	Stage IB	T2	M0	N0	unknow	Lung Basaloid Squamous Cell Carcinoma
TCGA-02-A52S	387	1	FEMALE	WHITE	57	Stage III	T4	MX	N2	YES	Lung Squamous Cell Carcinoma- Not Otherwise Specified (NOS)
TCGA-21-1075	2134	0	MALE	WHITE	57	Stage IIB	T2	M0	N1	unknow	Lung Squamous Cell Carcinoma- Not Otherwise Specified (NOS)
TCGA-39-5028	52	1	MALE	WHITE	75	Stage IIIA	T4	M0	N1	unknow	Lung Squamous Cell Carcinoma- Not Otherwise Specified (NOS)
TCGA-98-A53A	552	1	MALE	BLACK OR AFRICAN AMERICAN	70	Stage IB	T2a	M0	N0	NO	Lung Squamous Cell Carcinoma- Not Otherwise Specified (NOS)
TCGA-34-5232	2062	0	FEMALE	BLACK OR AFRICAN AMERICAN	75	Stage IIA	T1	M0	N1	unknow	Lung Squamous Cell Carcinoma- Not Otherwise Specified (NOS)
TCGA-85-8071	428	0	MALE	WHITE	52	Stage IIA	T1a	M0	N1	unknow	Lung Squamous Cell Carcinoma- Not Otherwise Specified (NOS)
TCGA-66-2766	31	0	MALE	unknow	54	Stage IIIA	T2	M0	N2	unknow	Lung Squamous Cell Carcinoma- Not Otherwise Specified (NOS)
TCGA-56-A5DS	8	0	FEMALE	WHITE	72	Stage IB	T2a	MX	N0	NO	Lung Squamous Cell Carcinoma- Not Otherwise Specified (NOS)
TCGA-NK-A5CX	111	0	MALE	WHITE	73	Stage IIA	T2b	MX	N0	NO	Lung Squamous Cell Carcinoma- Not Otherwise Specified (NOS)
TCGA-39-5016	2253	0	MALE	WHITE	44	Stage IIA	T2a	M0	N1	unknow	Lung Basaloid Squamous Cell Carcinoma
TCGA-52-7622	862	0	FEMALE	WHITE	62	Stage IA	T1a	M0	N0	unknow	Lung Squamous Cell Carcinoma- Not Otherwise Specified (NOS)
TCGA-60-2721	983	0	MALE	WHITE	73	Stage IB	T2	M0	N0	unknow	Lung Squamous Cell Carcinoma- Not Otherwise Specified (NOS)
TCGA-22-4599	1161	1	FEMALE	WHITE	73	Stage IB	T2a	M0	N0	unknow	Lung Squamous Cell Carcinoma- Not Otherwise Specified (NOS)
TCGA-77-8154	1092	0	MALE	unknow	67	Stage IA	T1	M0	N0	NO	Lung Squamous Cell Carcinoma- Not Otherwise Specified (NOS)
TCGA-02-A52N	1006	1	MALE	WHITE	78	Stage I	T2	MX	N0	NO	Lung Squamous Cell Carcinoma- Not Otherwise Specified (NOS)
TCGA-66-2782	365	1	MALE	unknow	71	Stage IIB	T3	M0	N0	unknow	Lung Squamous Cell Carcinoma- Not Otherwise Specified (NOS)
TCGA-22-1016	822	1	MALE	WHITE	65	Stage IB	T2	M0	N0	unknow	Lung Squamous Cell Carcinoma- Not Otherwise Specified (NOS)
TCGA-77-A5GH	713	0	MALE	unknow	81	Stage IB	T2a	M0	N0	unknow	Lung Squamous Cell Carcinoma- Not Otherwise Specified (NOS)

TCGA-77-7140	632	1	FEMALE	WHITE	69	Stage IIB	T2	M0	N1	unknow	Lung Squamous Cell Carcinoma- Not Otherwise Specified (NOS)
TCGA-18-3419	2811	0	MALE	unknow	73	Stage IIB	T2	M0	N1	unknow	Lung Squamous Cell Carcinoma- Not Otherwise Specified (NOS)
TCGA-22-5478	24	1	MALE	unknow	79	Stage IB	T2a	M0	N0	unknow	Lung Squamous Cell Carcinoma- Not Otherwise Specified (NOS)
TCGA-66-2734	1311	0	FEMALE	unknow	62	Stage IB	T2	M0	N0	unknow	Lung Squamous Cell Carcinoma- Not Otherwise Specified (NOS)
TCGA-18-3414	716	1	MALE	ASIAN	73	Stage IV	T4	M1	N1	unknow	Lung Squamous Cell Carcinoma- Not Otherwise Specified (NOS)
TCGA-43-6773	116	1	MALE	WHITE	76	Stage IIB	T2	MX	N1	unknow	Lung Squamous Cell Carcinoma- Not Otherwise Specified (NOS)
TCGA-21-1081	284	1	MALE	WHITE	69	Stage IIB	T2	M0	N1	unknow	Lung Squamous Cell Carcinoma- Not Otherwise Specified (NOS)
TCGA-37-3792	12	0	MALE	WHITE	69	Stage IB	T2	M0	N0	unknow	Lung Squamous Cell Carcinoma- Not Otherwise Specified (NOS)
TCGA-NC-A5HH	37	0	MALE	WHITE	53	Stage IA	T1	M0	N0	NO	Lung Basaloid Squamous Cell Carcinoma
TCGA-39-5019	1361	0	MALE	WHITE	70	Stage IB	T2a	M0	N0	unknow	Lung Squamous Cell Carcinoma- Not Otherwise Specified (NOS)
TCGA-94-8490	153	0	MALE	WHITE	70	Stage IIB	T3	M0	N0	NO	Lung Squamous Cell Carcinoma- Not Otherwise Specified (NOS)
TCGA-34-2609	951	1	MALE	BLACK OR AFRICAN AMERICAN	81	Stage IIB	T2	M0	N1	unknow	Lung Squamous Cell Carcinoma- Not Otherwise Specified (NOS)
TCGA-68-A59I	492	0	FEMALE	BLACK OR AFRICAN AMERICAN	73	Stage IIIA	T3	M0	N1	NO	Lung Squamous Cell Carcinoma- Not Otherwise Specified (NOS)
TCGA-37-4141	12	0	FEMALE	WHITE	65	Stage IA	T1b	M0	N0	unknow	Lung Squamous Cell Carcinoma- Not Otherwise Specified (NOS)
TCGA-34-2608	1000	1	MALE	WHITE	84	Stage IB	T2	M0	N0	unknow	Lung Squamous Cell Carcinoma- Not Otherwise Specified (NOS)
TCGA-60-2712	274	1	FEMALE	WHITE	79	Stage IIB	T2	M0	N1	unknow	Lung Squamous Cell Carcinoma- Not Otherwise Specified (NOS)
TCGA-56-7222	562	1	MALE	WHITE	60	Stage IB	T2a	M0	N0	unknow	Lung Squamous Cell Carcinoma- Not Otherwise Specified (NOS)
TCGA-77-A5G8	1290	0	MALE	unknow	70	Stage IIB	T3	M0	N0	unknow	Lung Squamous Cell Carcinoma- Not Otherwise Specified (NOS)
TCGA-63-A5MY	1052	0	MALE	unknow	63	Stage IA	T1b	M0	N0	NO	Lung Squamous Cell Carcinoma- Not Otherwise Specified (NOS)
TCGA-66-2786	790	0	FEMALE	unknow	68	Stage IA	T1	M0	N0	unknow	Lung Squamous Cell Carcinoma- Not Otherwise Specified (NOS)
TCGA-77-8150	1655	1	MALE	unknow	64	Stage IIIA	T3	M0	N1	YES	Lung Squamous Cell Carcinoma- Not Otherwise Specified (NOS)
TCGA-85-8351	510	0	MALE	WHITE	72	Stage IIA	T2a	M0	N1	unknow	Lung Squamous Cell Carcinoma- Not Otherwise Specified (NOS)
TCGA-21-1078	474	1	MALE	WHITE	77	Stage IB	T2	M0	N0	unknow	Lung Squamous Cell Carcinoma- Not Otherwise Specified (NOS)
TCGA-94-7943	143	0	MALE	WHITE	80	Stage IA	T1b	MX	NX	unknow	Lung Squamous Cell Carcinoma- Not Otherwise Specified (NOS)

TCGA-43-2578	550	0	FEMALE	WHITE	59	Stage IA	T1	M0	N0	unknow	Lung Squamous Cell Carcinoma- Not Otherwise Specified (NOS)
TCGA-66-2763	30	0	FEMALE	unknow	63	Stage IB	T2	M0	N0	unknow	Lung Squamous Cell Carcinoma- Not Otherwise Specified (NOS)
TCGA-98-8021	937	0	FEMALE	WHITE	75	Stage IA	T1a	M0	N0	NO	Lung Squamous Cell Carcinoma- Not Otherwise Specified (NOS)
TCGA-85-A4QR	600	0	MALE	WHITE	67	Stage IB	T2a	M0	N0	unknow	Lung Squamous Cell Carcinoma- Not Otherwise Specified (NOS)
TCGA-33-AASJ	3600	1	MALE	BLACK OR AFRICAN AMERICAN	60	Stage IB	T2	MX	N0	YES	Lung Squamous Cell Carcinoma- Not Otherwise Specified (NOS)
TCGA-22-1005	1953	1	MALE	WHITE	63	Stage IA	T1	M0	N0	unknow	Lung Squamous Cell Carcinoma- Not Otherwise Specified (NOS)
TCGA-22-5471	965	0	MALE	WHITE	75	Stage IB	T2	M0	N0	unknow	Lung Squamous Cell Carcinoma- Not Otherwise Specified (NOS)
TCGA-77-8156	10	0	MALE	unknow	60	Stage IB	T2a	M0	N0	unknow	Lung Squamous Cell Carcinoma- Not Otherwise Specified (NOS)
TCGA-39-5035	474	0	FEMALE	WHITE	72	Stage IA	T1b	M0	N0	unknow	Lung Squamous Cell Carcinoma- Not Otherwise Specified (NOS)
TCGA-60-2710	1714	0	FEMALE	WHITE	67	Stage IIA	T1	M0	N1	unknow	Lung Squamous Cell Carcinoma- Not Otherwise Specified (NOS)
TCGA-98-A538	826	0	MALE	WHITE	67	Stage IIB	T3	M0	N0	NO	Lung Squamous Cell Carcinoma- Not Otherwise Specified (NOS)
TCGA-58-8390	911	0	MALE	WHITE	70	Stage IIA	T2b	M0	N0	NO	Lung Squamous Cell Carcinoma- Not Otherwise Specified (NOS)
TCGA-43-7656	315	0	MALE	WHITE	71	Stage IA	T1b	MX	N0	unknow	Lung Squamous Cell Carcinoma- Not Otherwise Specified (NOS)
TCGA-22-4591	623	1	MALE	WHITE	80	Stage IIIA	T3	M0	N2	unknow	Lung Squamous Cell Carcinoma- Not Otherwise Specified (NOS)
TCGA-37-A5EL	1143	1	MALE	WHITE	53	Stage IIB	T3	M0	N0	NO	Lung Squamous Cell Carcinoma- Not Otherwise Specified (NOS)
TCGA-77-8138	539	1	MALE	unknow	74	Stage IB	T2	M0	N0	YES	Lung Squamous Cell Carcinoma- Not Otherwise Specified (NOS)
TCGA-66-2744	30	0	MALE	unknow	71	Stage IIB	T2	M0	N1	unknow	Lung Squamous Cell Carcinoma- Not Otherwise Specified (NOS)
TCGA-21-5787	329	1	MALE	BLACK OR AFRICAN AMERICAN	65	Stage IIIA	T2	M0	N2	unknow	Lung Squamous Cell Carcinoma- Not Otherwise Specified (NOS)
TCGA-NK-A7XE	13	0	MALE	BLACK OR AFRICAN AMERICAN	66	Stage IIIB	T4	M0	N2	NO	Lung Squamous Cell Carcinoma- Not Otherwise Specified (NOS)
TCGA-34-5239	707	0	MALE	WHITE	75	Stage IIIA	T4	M0	N0	unknow	Lung Squamous Cell Carcinoma- Not Otherwise Specified (NOS)
TCGA-60-2716	1356	0	MALE	unknow	39	Stage IIB	T2	M0	N1	unknow	unknow
TCGA-60-2719	932	0	FEMALE	WHITE	83	Stage IB	T1	M0	N0	unknow	Lung Squamous Cell Carcinoma- Not Otherwise Specified (NOS)
TCGA-94-7557	5	1	MALE	BLACK OR AFRICAN AMERICAN	73	Stage IB	T2	M0	N0	unknow	Lung Squamous Cell Carcinoma- Not Otherwise Specified (NOS)

TCGA-56-8305	105	0	MALE	WHITE	72	Stage IB	T2a	M0	N0	NO	Lung Squamous Cell Carcinoma- Not Otherwise Specified (NOS)
TCGA-66-2742	641	0	MALE	unknow	70	Stage IV	T2	M1	N1	unknow	Lung Squamous Cell Carcinoma- Not Otherwise Specified (NOS)
TCGA-60-2713	1580	0	MALE	WHITE	64	Stage IB	T2	M0	N0	unknow	Lung Squamous Cell Carcinoma- Not Otherwise Specified (NOS)
TCGA-02-A5IB	340	1	FEMALE	WHITE	71	Stage III	T3	MX	N1	YES	Lung Squamous Cell Carcinoma- Not Otherwise Specified (NOS)
TCGA-43-8118	89	1	FEMALE	WHITE	55	Stage IA	T1b	M0	N0	NO	Lung Squamous Cell Carcinoma- Not Otherwise Specified (NOS)
TCGA-LA-A7SW	408	1	MALE	BLACK OR AFRICAN AMERICAN	71	Stage IIIA	T3	MX	N1	unknow	Lung Squamous Cell Carcinoma- Not Otherwise Specified (NOS)
TCGA-63-A5ML	1386	0	MALE	unknow	68	Stage IB	T2	M0	N0	NO	Lung Squamous Cell Carcinoma- Not Otherwise Specified (NOS)
