# Supplementary material for: DNA damage repair gene signature model for predicting prognosis and chemotherapy outcomes in lung squamous cell carcinoma
Source: BMC Cancer. 2022 Aug 8;22:866. doi: 10.1186/s12885-022-09954-x (PMC9361681; doi:10.1186/s12885-022-09954-x)
Supplement: Supplementary file 1 — Additional file 1: Table S1. Clinical information. Table S2. Marker For ssGSEA. Table S3. Prognosis related DNA repair genes. Table S4. Differently expressed genes. [file 12885_2022_9954_MOESM1_ESM.zip › Table S2. Marker For ssGSEA_ESM.pdf]

## Additional file table S2 marker For ssGSEA

### The annotation of 16 immune cells in ssGSEA

| aDCs | B_cells | CD8+T_cells | DCs | iDCs | Macrophages | Mast_cells | TIL | | | | | Treg | | |
|---|---|---|---|---|---|---|---|---|---|---|---|---|---|---|
| CD83 | BACH2 | CD8A | CCL17 | CD1A | C11orf45 | CMA1 | ITM2C | LILRB1 | TNFRSF4 | NCF4 | TBC1D10C | IL12RB2 | ANKRD10 | ETV7 |
| LAMP3 | BANK1 | | CCL22 | CD1E | CD68 | MS4A2 | CD38 | DOK2 | CD79A | DOCK2 | GVINP1 | TMPRSS6 | CD274 | BCL2L1 |
| CCL1 | BLK | | CD209 | | CLEC5A | TPSAB1 | THEMIS2 | CD6 | CD247 | CD8A | P2RY8 | CTSC | CASP1 | RRAGB |
| | BTLA | | CCL13 | | CYBB | | GLYR1 | PAG1 | LCP2 | CCR7 | VAMP5 | LAPTM4B | LY75 | ACSL4 |
| | CD79A | | | | FUCA1 | | ICOS | LAX1 | CD3D | DOCK11 | KLRK1 | TFRC | NPTN | CHRNA6 |
| | CD79B | | | | GPNMB | | F5 | PLEK | CD27 | PARVG | | RNF145 | SSTR3 | BATF |
| | FCRL1 | | | | HS3ST2 | | TIGIT | PIK3CD | SH2D1A | SPNS1 | | NETO2 | GRSF1 | LAX1 |
| | FCRL3 | | | | LGMN | | KLRD1 | SLAMF1 | FYB | CD52 | | ADAT2 | CSF2RB | ADPRH |
| | HVCN1 | | | | MMP9 | | IRF4 | XCL1 | ARHGAP30 | HCLS1 | | CHST2 | TMEM184C | TNFRSF4 |
| | RALGPS2 | | | | TM4SF19 | | FCRL5 | GPR171 | ACAP1 | ARHGAP9 | | NFE2L3 | NDFIP2 | CHST7 |
| | | | | | | | SIRPG | XCL2 | CST7 | SELL | | LIMA1 | ZBTB38 | NFAT5 |
| | | | | | | | LPXN | TBX21 | CD3G | MPEG1 | | IL1R2 | ERI1 | ZNF282 |
| **T_helper_cells** | **Tfh** | **Th1_cells** | **Th2_cells** | **NK_cells** | **pDCs** | **Neutrophils** | IL2RG | CD2 | IL2RB | TRAT1 | | ICOS | TRAF3 | ENTPD1 |
| CD4 | PDCD1 | IFNG | PMCH | KLRC1 | CLEC4C | EVI2B | CCL5 | CD53 | CD3E | IL10RA | | HSDL2 | NAB1 | TNFRSF18 |
| | CXCL13 | TBX21 | LAIR2 | KLRF1 | CXCR3 | HSD17B11 | LCK | KLHL6 | FCRL3 | PAX5 | | HTATIP2 | HS3ST3B1 | METTL7A |
| | CXCR5 | CTLA4 | SMAD2 | | GZMB | KDM6B | TRAF3IP3 | SLAMF6 | CORO1A | EVI2B | | FKBP1A | LAYN | KSR1 |
| | | STAT4 | CXCR6 | | IL3RA | MEGF9 | CD86 | CD40 | LY9 | 44080 | | TIGIT | JAK1 | SSH1 |
| | | CD38 | GATA3 | | IRF7 | MNDA | MAL | SIT1 | CD48 | CD28 | | CCR8 | VDR | CADM1 |
| | | IL12RB2 | IL26 | | IRF8 | NLRP12 | ITK | CCR2 | HCST | STAT4 | | LTA | LEPROT | IL1R1 |
| | | LTA | | | LILRA4 | PADI4 | TCL1A | PTPRC | PTPRCAP | GIMAP6 | | SLC35F2 | GCNT1 | ACP5 |
| | | CSF2 | | | PHEX | SELL | CYBB | PLAC8 | SASH3 | PRKCB | | IL21R | PTPRJ | THADA |
| | | | | | PLD4 | TRANK1 | CSF2RB | NCKAP1L | ARHGAP25 | MS4A1 | | AHCYL1 | IKZF2 | CD177 |
| | | | | | PTCRA | VNN3 | IKZF1 | IL7R | LAT | GPR18 | | SOCS2 | CSF1 | MAGEH1 |

### The annotation of 13 functions in ssGSEA

| APC costimulation | Inflammation promoting | T cell costimulation | T cell coinhibition | Type I IFN Reponse | HLA | Parainflammation | | Checkpoint | | CCR | | | |
|---|---|---|---|---|---|---|---|---|---|---|---|---|---|
| CD40 | CCL5 | CD2 | BTLA | DDX4 | HLA-E | CXCL10 | RETNLB | IDO1 | CD274 | CCL16 | TNFRSF18 | CX3CL1 | IL6R |
| CD58 | CD19 | CD226 | C10orf54 | IFIT1 | HLA-DPB2 | PLAT | IFIT2 | LAG3 | HAVCR2 | TPO | IL17RD | BMP5 | BMPR2 |
| CD70 | CD8B | CD27 | CD160 | IFIT2 | HLA-C | CCND1 | ISG15 | CTLA4 | CD27 | TGFBR2 | IL17D | CXCR2 | IFNE |
| ICOSLG | CXCL10 | CD28 | CD244 | IFIT3 | HLA-J | LGMN | OAS2 | TNFRSF9 | BTLA | CXCL2 | IL27 | TNFRSF10D | IL1RAPL2 |
| SLAMF1 | CXCL13 | CD40LG | CD274 | IRF7 | HLA-DQB1 | PLAUR | REL | ICOS | LGALS9 | CCL14 | CCL7 | BMP2 | IL3RA |
| TNFSF14 | CXCL9 | ICOS | CTLA4 | ISG20 | HLA-DQB2 | AIM2 | OAS3 | CD80 | TMIGD2 | TGFBR3 | IL1R1 | CXCL14 | BMP4 |
| TNFSF15 | GNLY | SLAMF1 | HAVCR2 | MX1 | HLA-DQA2 | MMP7 | CD44 | PDCD1LG2 | CD28 | IL11RA | CXCR4 | CCL28 | CCL24 |
| TNFSF18 | GZMB | TNFRSF18 | LAG3 | MX2 | HLA-DQA1 | ICAM1 | PPARG | TIGIT | CD48 | CCL11 | CXCR2P1 | CXCL3 | TNFSF13B |
| TNFSF4 | IFNG | TNFRSF25 | LAIR1 | RSAD2 | HLA-A | MX2 | BST2 | CD70 | TNFRSF25 | IL4I1 | TGFB1I1 | BMP6 | CCR4 |
| TNFSF8 | IL12A | TNFRSF4 | TIGIT | TNFSF10 | HLA-DMA | CXCL9 | OAS1 | TNFSF9 | CD40LG | IL33 | IFNGR1 | CCL21 | IL2RA |
| TNFSF9 | IL12B | TNFRSF8 | | | HLA-DOB | ANXA1 | NOX1 | ICOSLG | ADORA2A | CXCL12 | IL9R | CXCL9 | IL32 |
| | IRF1 | TNFRSF9 | | | HLA-DRB1 | TLR2 | PLA2G2A | KIR3DL1 | VTCN1 | CXCL10 | IL1RAPL1 | CCL23 | TNFRSF10C |
| | PRF1 | TNFSF14 | | | HLA-H | PLA2G2D | IFIT1 | CD86 | CD160 | BMPER | IL11 | IL6 | IL22RA1 |
| | STAT1 | | | | HLA-B | ITGA2 | IFITM3 | PDCD1 | CD44 | BMP8A | CSF1 | IFNL1 | BMPR1A |
| | TBX21 | | | | HLA-DRB5 | MX1 | IL1RN | LAIR1 | TNFSF18 | CXCL11 | IL20RA | IL16 | CXCR5 |
| | | | | | HLA-DOA | HMOX1 | | TNFRSF8 | TNFRSF18 | IL21R | IL25 | IL1RL1 | CXCR3 |
| | | | | | HLA-DPB1 | CD276 | | TNFSF15 | BTNL2 | IL17B | TNFRSF4 | ILK | IFNA8 |
| **Cytolytic activity** | **MHC class I** | **Type II IFN Reponse** | **APC coinhibition** | | HLA-DRA | TIRAP | | TNFRSF14 | C10orf54 | TNFRSF9 | IL18 | CCL25 | IL17REL |
| PRF1 | B2M | GPR146 | C10orf54 | | HLA-DRB6 | IL33 | | IDO2 | CD200R1 | ILF2 | ILF3 | ILDR2 | IFNB1 |
| GZMA | HLA-A | SELP | CD274 | | HLA-L | PTGES | | CD276 | TNFSF4 | CX3CR1 | CCL20 | CXCR1 | IFNAR1 |
| | TAP1 | AHR | LGALS9 | | HLA-F | TNFRSF12A | | CD40 | CD200 | CCR8 | TNFRSF12A | IL36RN | TNFRSF1B |
| | | | PDCD1LG2 | | HLA-G | SCARB1 | | TNFRSF4 | NRP1 | TNFSF12 | IL6ST | IL34 | CCL17 |
| | | | PVRL3 | | HLA-DMB | CD14 | | TNFSF14 | | CSF3 | CXCL13 | TGFB1 | IL19 |
| | | | | | HLA-DPA1 | BLNK | | HHLA2 | | TNFSF4 | IL12B | IFNG | |
| | | | | | | IFIT3 | | CD244 | | BMP3 | TNFRSF8 | ILKAP | |
