# Supplementary material for: DNA damage repair gene signature model for predicting prognosis and chemotherapy outcomes in lung squamous cell carcinoma
Source: BMC Cancer. 2022 Aug 8;22:866. doi: 10.1186/s12885-022-09954-x (PMC9361681; doi:10.1186/s12885-022-09954-x)
Supplement: Supplementary file 1 — Additional file 1: Table S1. Clinical information. Table S2. Marker For ssGSEA. Table S3. Prognosis related DNA repair genes. Table S4. Differently expressed genes. [file 12885_2022_9954_MOESM1_ESM.zip › Table S3. Prognosis related DNA repair genes_ESM.pdf]

**Additional file table S3 Prognosis related DNA repair genes**

| Gene | coef | HR | HR.95L | HR.95H | pvalue |
|---|---|---|---|---|---|
| POLD4 | 0.281777 | 1.325483 | 0.989875 | 1.774876 | 0.058538 |
| MRPL40 | -0.41386 | 0.661093 | 0.511536 | 0.854376 | 0.001563 |
| ITPA | 0.611709 | 1.843579 | 1.3163 | 2.582073 | 0.000372 |
| ERCC3 | -0.385 | 0.680451 | 0.450581 | 1.027592 | 0.067168 |
| TK2 | 0.475033 | 1.608067 | 1.060213 | 2.439018 | 0.025413 |
| POLR3GL | -0.70179 | 0.495697 | 0.353633 | 0.694831 | 0.0000464 |
| VPS28 | 0.415677 | 1.515397 | 1.100772 | 2.086198 | 0.010814 |
| CANT1 | 0.433727 | 1.542997 | 1.118992 | 2.127666 | 0.00815 |
| SDCBP | 0.194292 | 1.21445 | 0.940948 | 1.56745 | 0.135588 |
| CCNO | -0.2904 | 0.747963 | 0.612321 | 0.913652 | 0.004448 |
