# Supplementary material for: DNA damage repair gene signature model for predicting prognosis and chemotherapy outcomes in lung squamous cell carcinoma
Source: BMC Cancer. 2022 Aug 8;22:866. doi: 10.1186/s12885-022-09954-x (PMC9361681; doi:10.1186/s12885-022-09954-x)
Supplement: Supplementary file 1 — Additional file 1: Table S1. Clinical information. Table S2. Marker For ssGSEA. Table S3. Prognosis related DNA repair genes. Table S4. Differently expressed genes. [file 12885_2022_9954_MOESM1_ESM.zip › Table S4. Differently expressed genes_ESM.pdf]

Additional file table S4. Differently expressed genes.

Gene	Normal Mean	Tumor Mean	logFC	pValue	fdr
RFC4	1.504281	4.213879	2.709598	9.67E-31	5.65E-29
ZWINT	1.6099	4.385617	2.775717	1.34E-30	5.65E-29
RAD51	0.557732	2.660823	2.103091	1.39E-30	5.65E-29
POLR2H	3.062739	4.93347	1.870732	1.67E-30	5.65E-29
RAE1	2.147522	3.462606	1.315085	1.99E-30	5.65E-29
FEN1	2.412673	4.49931	2.086637	2.34E-30	5.65E-29
AK1	3.738018	1.77489	-1.96313	3.73E-30	7.72E-29
RFC5	1.900455	3.394162	1.493707	6.33E-30	1.04E-28
PCNA	4.817272	6.709564	1.892292	6.47E-30	1.04E-28
NCBP2	3.649865	4.979858	1.329993	2.13E-29	2.69E-28
NELFCD	3.161475	4.329387	1.167912	2.15E-29	2.69E-28
RFC2	3.226476	4.52769	1.301214	2.22E-29	2.69E-28
NME1	2.325295	4.424161	2.098866	2.45E-29	2.73E-28
TYMS	1.796649	3.989985	2.193336	2.72E-29	2.82E-28
HPRT1	3.560978	5.197793	1.636815	3.06E-29	2.96E-28
ALYREF	4.487579	5.832431	1.344851	2.14E-28	1.94E-27
RFC3	1.831061	3.056201	1.22514	3.06E-28	2.61E-27
UMPS	1.960692	3.095022	1.134329	3.43E-28	2.76E-27
SSRP1	4.287103	5.298298	1.011195	5.66E-28	4.32E-27
SAC3D1	1.868984	3.044364	1.17538	7.19E-28	5.21E-27
LIG1	1.843795	3.089076	1.245281	8.15E-28	5.63E-27
POLA2	1.756588	2.798112	1.041524	2.62E-27	1.73E-26
SDCBP	6.29114	5.122239	-1.1689	2.99E-27	1.89E-26
ADRM1	5.02324	6.035893	1.012654	3.91E-27	2.36E-26
DGUOK	4.288921	5.290109	1.001188	5E-27	2.79E-26
POLD1	2.296143	3.303253	1.007109	2.63E-24	1.23E-23
POLB	2.032019	3.105549	1.07353	4.7E-24	2.06E-23
NME4	3.372978	4.478467	1.105488	5.43E-24	2.25E-23
PRIM1	1.575971	2.645728	1.069758	1.28E-23	5.17E-23
UPF3B	2.250756	3.276362	1.025606	3.47E-23	1.33E-22
ADA	1.688358	3.179619	1.491261	7.87E-21	2.48E-20
VPS37D	0.71976	1.741286	1.021526	4.48E-20	1.3E-19
CCNO	0.86038	1.897255	1.036875	5.02E-14	9.84E-14
BCAM	6.083002	5.00588	-1.07712	1.13E-10	1.87E-10

logFC: log2 fold change. fdr: false discovery rate.
